# Applying appropriate frequency criteria to advance acoustic behavioural guidance systems for fish

**DOI:** 10.1038/s41598-023-33423-5

**Published:** 2023-05-18

**Authors:** A. Holgate, P. R. White, T. G. Leighton, P. S. Kemp

**Affiliations:** 1 International Centre for Ecohydraulics Research, Faculty of Engineering and Physical Sciences, University of Southampton, Southampton, UK; 2 Institute of Sound and Vibration Research, Faculty of Engineering and Physical Sciences, University of Southampton, Southampton, UK

**Keywords:** Ecology, Zoology, Ecology

## Abstract

Deterrents that use acoustics to guide fish away from dangerous areas depend on the elicitation of avoidance in the target species. Acoustic deterrents select the optimum frequency based on an assumption that highest avoidance is likely to occur at the greatest sensitivity. However, such an assumption may be unfounded. Using goldfish (*Carassius auratus*) as a suitable experimental model, this study tested this as a null hypothesis. Under laboratory conditions, the deterrence thresholds of individual goldfish exposed to 120 ms tones at six frequencies (250–2000 Hz) and four Sound Pressure Levels (SPL 115–145 dB) were quantified. The deterrence threshold defined as the SPL at which 25% of the tested population startled was calculated and compared to the hearing threshold obtained using Auditory Evoked Potential and particle acceleration threshold data. The optimum frequency to elicit a startle response was 250 Hz; different from the published hearing and particle acceleration sensitivities based on audiograms. The difference between the deterrence threshold and published hearing threshold data varied from 47.1 dB at 250 Hz to 76 dB at 600 Hz. This study demonstrates that information obtained from audiograms may poorly predict the most suitable frequencies at which avoidance behaviours are elicited in fish.

## Introduction

As the most threatened ecosystems on the planet^1^ and facing greater environmental pressures than any other^2^, fresh waters are experiencing a ‘biodiversity crisis’^3^. Freshwater vertebrates, which constitute a third of the world’s total^4^, are suffering population declines at twice the rate of those observed in marine or terrestrial ecosystems^2,3^. There are multiple explanations for the rapid^3^ and widespread deterioration of freshwater environments, including habitat degradation and loss, such as wetland drainage^5^; channelization^6^; invasive species^7^; climate change^8^; changing land use (e.g. increased agricultural production^9^); reduced river flows^10^; declining water quality^11^; novel pollutants (e.g. microplastics^12^; anthropogenic noise^13^; artificial light at night^14^); and the cumulative effects of multiple stressors^2,15^. Centuries of river engineering, in particular, has severely damaged freshwater ecosystems, with many rivers channelised and impounded, reducing longitudinal, lateral and vertical connectivity and fragmenting fluvial habitat^16^.

Regeneration of freshwater ecosystems requires the integration of social, financial, legal and technological approaches^2,17^. Focusing on technology, there are a range of environmental impact mitigation solutions that may partially ameliorate some of the worst effects of river engineering^16^. For example, fish passes are designed to enable fish to negotiate river infrastructure, such as weirs and dams^18^, while physical screens are intended to prevent entrainment of fish into water intakes (e.g. at hydropower stations or cooling water systems), and in some instances to guide them to alternative routes, such as bypass systems^19^. The efficacy of environmental impact mitigation technology can be highly variable with site, context, and species^20^, as illustrated for both fish passes^21^ and physical screens, which themselves can be damaging if poorly designed^22,23^. Recently, efforts have been directed at improving the effectiveness of this technology. This has included a return to fundamental first principles and a reductionist approach to better understand the mechanisms that determine efficiency (e.g. influence of hydrodynamics^24^; species^25^; personality^26^). At the same time, some have sought to take a more applied approach to combine technologies with the hope that efficiency will be improved if synergistic effects are realised (e.g. acoustics and light^27^). In some cases, behavioural deterrents have been developed with the view to using them in combination with traditional physical screening systems to enhance overall screening and guidance efficiency (e.g. application of the marginal gains concept to fish screening^28,29^).

Behavioural guidance systems take advantage of the innate behaviours, typically aversive (akin to an anti-predator like escape reaction^13,30^), elicited by fish in response to a stimulus resulting in modification of the movement trajectories of a target species. A wide variety of stimuli, mostly abiotic, have been employed as deterrents, including electricity^31^, strobe lights^32^ and acoustics (e.g. experimentally^28,33^ and in situ^34^). Sound can be advantageous in some contexts because the stimulus is omnidirectional, and so can simultaneously reach a number of target individuals within a locality and is not affected by changes in illumination or turbidity as are those that are mediated through vision. As a consequence, acoustic deterrents have been widely employed to reduce fish mortality at water abstraction points^35^, or to control the spread of invasive species (e.g. bighead carp, *Hypophthalmichthys nobilis*, in the Great Lakes, USA^36^).

A limitation of acoustic deterrents is that their effectiveness can be highly variable^37–39^. For example, deterrence efficiency has been observed to range from 5 to 90% for juvenile Chinook salmon^40^ (*Oncorhynchus tshawytscha;* under experimental conditions) and from 11^41^ to 87.9%^35^ for European Sprat (*Sprattus sprattus*). There are many proposed explanations for such inconsistent efficiencies, including the use of acoustic cues with frequencies outside the hearing range of the target species^42^, or because the ambient sound levels were not accounted for^39^. Alternatively, the results may reflect a lack of consideration of the relationship between hearing abilities, defined by the frequency range of the stimulus and hearing sensitivity of the individual, and the behavioural response of the target species^39^. Whilst it is impossible to know the hearing and deterrence thresholds of every individual fish, if we could quantify the intraspecific variation observed in the population, we could design deterrents that meet conservation targets^43^. In the past it was assumed that, for different frequencies, the difference between the hearing threshold and the sound pressure level (SPL) that elicits a behavioural response is uniform, and that this difference may be expressed in dB above the hearing threshold (dB_ht_)^44^. This logic suggests that, for a fixed stimulus level, the frequency that is most likely to engender a response is the one at which the animal’s hearing is most sensitive, i.e. where the sensory threshold is lowest. Such an assumption is not evidence-based and has been dismissed among some researchers^45,46^. Nevertheless, some industry practitioners utilise the most sensitive hearing level to predict the behavioural response of fish when developing behavioural deterrents, including the use of audiograms in the design of deterrents for invasive carp^47^. To advance the next generation of acoustic deterrents that may be used in combination with other, perhaps more traditional environmental impact mitigation technology, there is a need to return to fundamental first principles.

This experimental study examined the relationship between frequency of hearing sensitivity and the deterrence thresholds of fish. Goldfish (*Carassius auratus*) were selected as the model species due to the large amount of physiologically derived information available on their hearing capabilities^48,49^ and their ease of maintenance under laboratory conditions. This study used the startle response exhibited by fish when exposed to an acoustic stimulus as a proxy for a deterrent response, enabling definition of a threshold to inform acoustic deterrence (as opposed to a behavioural hearing threshold).

This study determined the: (1) presence and absence of startle responses to determine the proportion of the population that exhibited avoidance and, then, the relationship between probability of startling and frequency (250; 400; 600; 800; 1000; 2000 Hz) and SPL (115; 125; 135; 145 dB re 1 μPa); (2) deterrence threshold for each frequency defined as the SPL at which at least 25% of the sampled population elicited a startle response, a proxy for deterrence; and (3) relationship between the deterrence threshold, the hearing threshold, and the particle acceleration (PA) threshold at each frequency based on existing data obtained from audiograms for the subject species.

## Results

**Figure 1:**
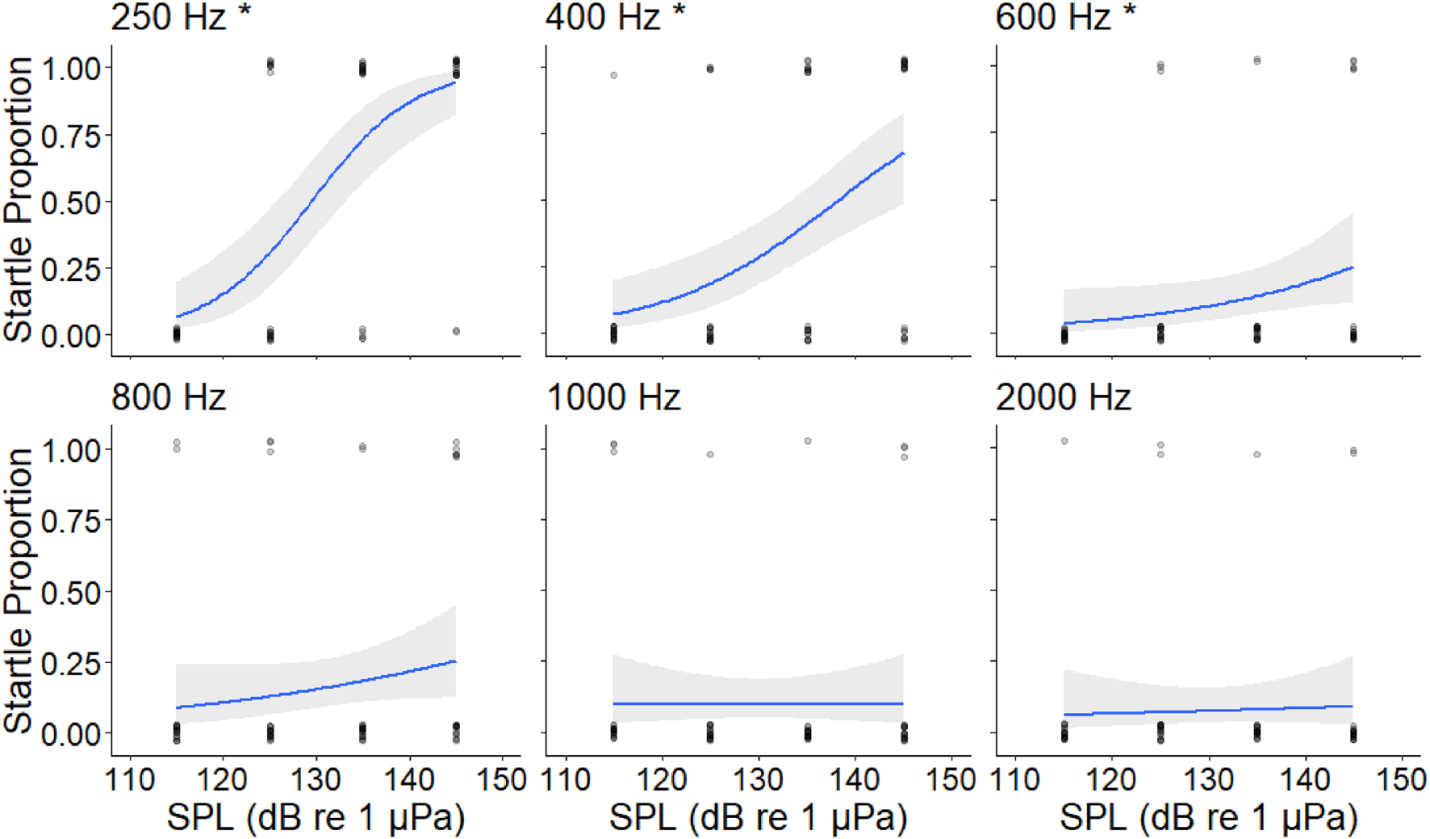
Logistic regression curves illustrating the proportion of goldfish that startled in response to a 120 ms pure tone (115; 125; 135; 145 dB re 1 μPa). The asterisk (*) denotes significance (p < 0.05). Results were plotted for each frequency (250; 400; 600; 800; 1000; 2000 Hz). The grey regions indicate 95% confidence intervals.

Startles were observed in all frequency treatments. The probability (Fig. 1) of startling decreased with frequency (*z* = 6.084, *p* ≤ 0.001), and within each frequency treatment increased with SPL for 250 Hz (*z* = 4.886, *p* ≤ 0.001), 400 Hz (*z* = 3.925, *p* ≤ 0.001) and 600 Hz (*z* = 2.003, *p* = 0.0452), but not for 800 Hz (*z* = 1.463, *p* = 0.144), 1000 Hz (*z* = 0, *p* = 1) or 2000 Hz (*z* = 0.379, *p* = 0.705).

**Figure 2:**
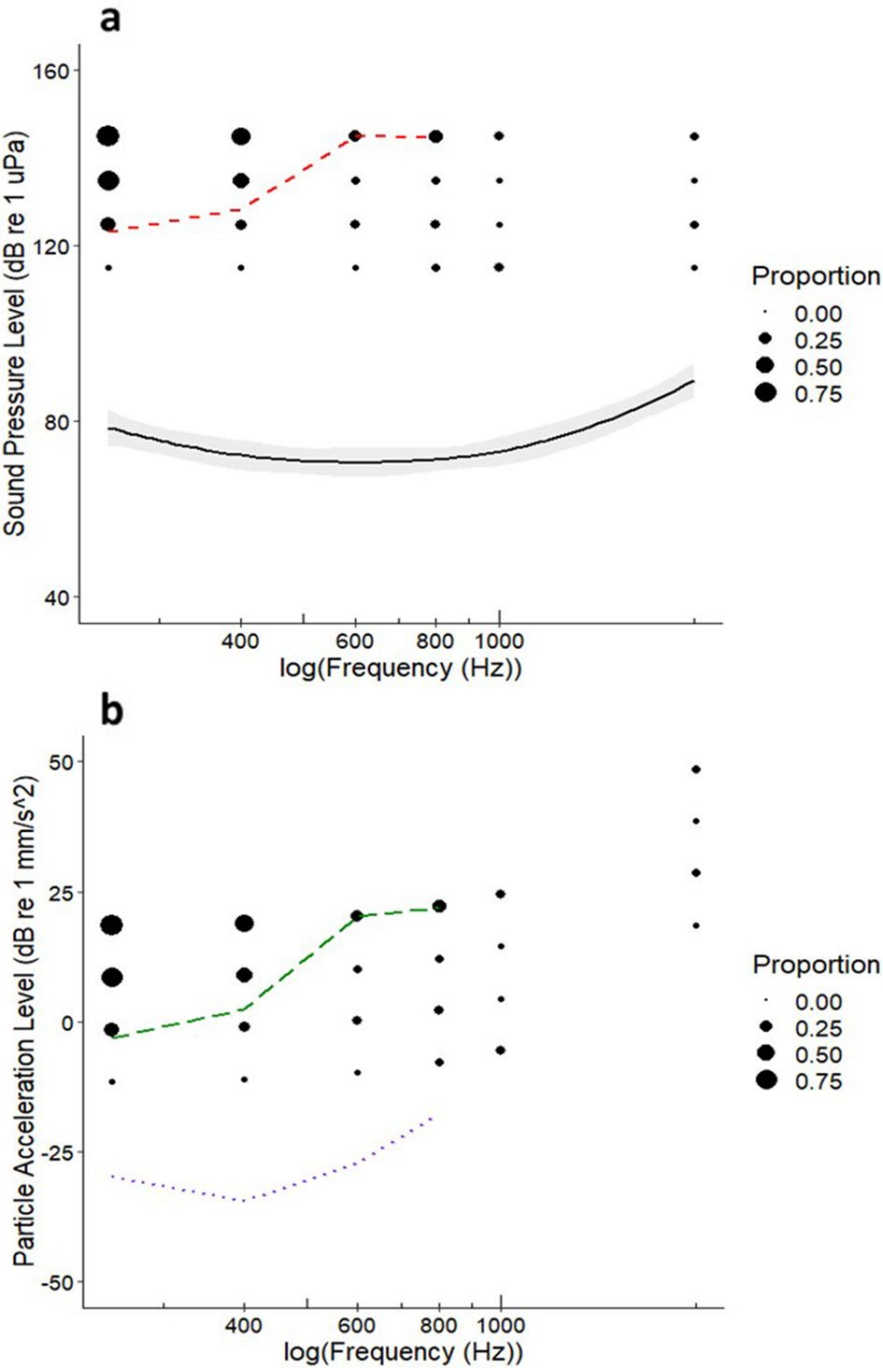
The deterrence threshold for (**a**) (dashed line) indicates the sound pressure level and for (**b**) (long-dashed line) indicated the particle acceleration level for each frequency treatment at which 25% of the test population responded to a 120 ms pure tone stimulus. The solid circles indicate the proportion of the test population that startled. Hearing sensitivity thresholds based on the published literature are indicated by (**a**) the Auditory Evoked Potential, AEP, (solid line) and (**b**) the particle acceleration level (dotted line). The shaded area indicates the 95% confidence interval.

At least 25% of the test population startled at frequencies that ranged from 250 to 800 Hz. A higher SPL (Fig. 2a) and PA (Fig. 2b) was required to elicit a response for 25% of the population at 600 Hz and 800 Hz, compared to 250 Hz and 400 Hz. The published hearing sensitivity threshold for SPL had a minimum at ≈ 600 Hz and at ≈ 400 Hz for PA, after which the hearing threshold subsequently increased with frequency. The SPL eliciting a startle response for 25% of the population at 250, 400, 600, and 800 Hz was 123, 128, 145 and 145 dB re 1 μPa, respectively. The PA eliciting a startle response for 25% of the population at 250, 400, 600, and 800 Hz was − 3, 2, 20 and 22 dB re 1 mm s^−2^, respectively.

**Figure 3:**
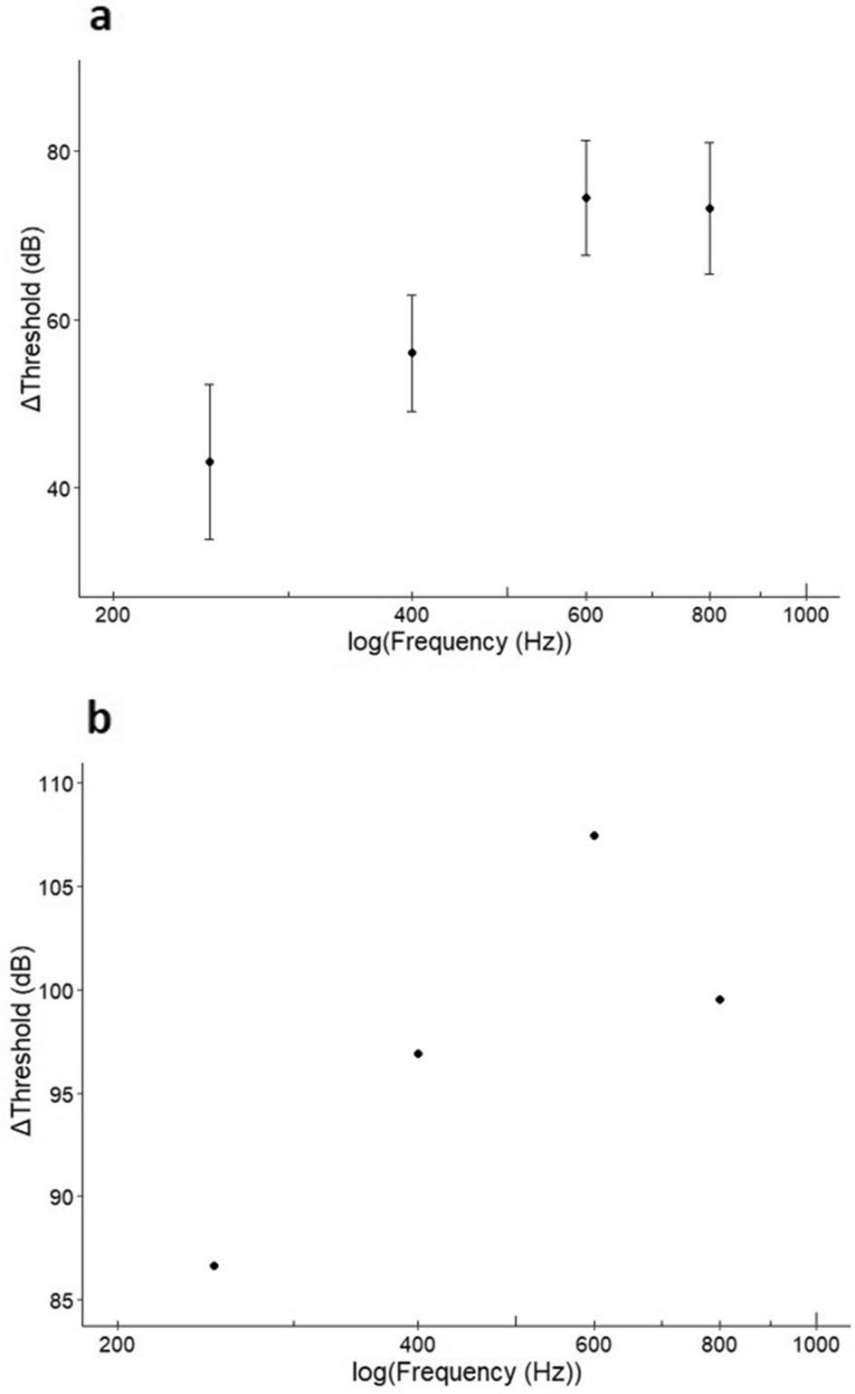
The SPL *ΔThreshold* between the 25% deterrence threshold and the (**a**) mean Auditory Evoked Potential (AEP) hearing threshold (± SD) and (**b**) the particle acceleration threshold (N = 1, therefore SD was not included) from previously published data of goldfish at 250 Hz; 400 Hz; 600 Hz; 800 Hz.

The *ΔThreshold* for SPL increased with frequency, i.e. Auditory Evoked Potential (AEP) (Fig. 3a; *t* = 8.367, *p* ≤ 0.001). The small sample size for the PA *ΔThreshold* meant only visual inspection was conducted. Visual inspection of the plot (Fig. 3b) suggested the *ΔThreshold* increased with frequency up to 600 Hz but decreased at 800 Hz. The SPL *ΔThreshold* for 250, 400, 600 and 800 Hz was 43, 56, 74 and 73 dB comparing the deterrence threshold to the AEP hearing threshold. When comparing the deterrence threshold to the PA threshold, the *ΔThreshold* was 52, 62, 80 and 76 dB for 250, 400, 600 and 800 Hz, respectively. A priori contrasts (Table 1) revealed the *ΔThreshold* at 250 Hz was less than all other frequencies (*p* < 0.001), and the *ΔThreshold* at 400 Hz was lower compared to 600 and 800 Hz (*p* < 0.001).

**Table 1:** Planned orthogonal contrasts between *ΔThreshold* at four frequencies (250, 400, 600, 800 Hz). *ΔThreshold* is the difference between deterrence threshold obtained in this study and existing published hearing threshold data for goldfish.

Contrast	Effect of group	
	*z*	*p*
250 Hz vs 400 Hz	− 3.759	< 0.001
250 Hz vs 600 Hz	− 10.558	< 0.001
250 Hz vs 800 Hz	− 8.773	< 0.001
400 Hz vs 600 Hz	− 6.912	< 0.001
400 Hz vs 800 Hz	− 5.097	< 0.001

## Discussion

A common assumption in the design of fish deterrents is that the frequency of greatest hearing sensitivity corresponds with that most likely to elicit an avoidance response in the species of interest^47^. Using a well-studied model species (goldfish) for which considerable information on hearing capability is available^48,49^, and the startle reaction as a proxy measure of deterrence, we found no evidence to support this assumption. Instead, one-quarter of all fish tested in the 250 Hz treatment startled in response to an acoustic stimulus of 123 dB re 1 μPa. This frequency is lower than that at which hearing is most sensitive, i.e. approx. 600 Hz (Supplementary Table S1); a frequency at which a higher SPL of 145 dB re 1 μPa was required to achieve an equivalent proportion of startles. This finding suggests that design criteria for behavioural deterrents should be reviewed and further advanced by returning to first principles to determine the characteristics of the sound field, including frequency and SPL, that are most likely to induce avoidance behaviour as required to meet efficiency targets.

Comparisons between hearing and deterrence thresholds in fish are limited. There are few examples of attempts to define deterrence thresholds, with the exception of those used to determine the hearing ability of larval fishes^50,51^. Turning to the wider biology and audiology literature, however, it is apparent that there is a wealth of valuable knowledge, insight and methodologies that could be usefully applied to freshwater bioacoustics^43^. For example, a comparison between the hearing threshold derived from audiograms of rats (*Rattus norvegicus*) and deterrence thresholds indicated that the two ran parallel when plotted against frequency on a logarithmic dB scale^52^, in contradiction to the results of our study. In humans, however, the thresholds of hearing and loudness discomfort, a measure of behavioural intolerance, often occur at dissimilar frequencies^53^ i.e., the usable dynamic range (the dB difference between the hearing threshold and the threshold for some adverse reaction) is not independent of frequency. Towards the high frequency upper limit of human hearing, there is evidence that this usable dynamic range becomes so small that adverse effects are seen in some individuals at the lowest SPLs at which they can perceive the sound^54–58^. Additionally, humans sometimes finding irritating noise (e.g., hums ranging from about 10 Hz to 200 Hz^59^) at frequencies lower than those at which hearing is most sensitive.

The fish observed in this study were more responsive at the lower frequencies within their hearing range. Our findings complement those of another study that investigated the behavioural reaction threshold of fish in situ, focusing on marine species in the context of impacts of anthropogenic noise^60^. In agreement with our findings, these authors also observed that the difference between hearing and reaction thresholds varied with frequency. However, they found that the eight subject species they studied reacted very differently, making generalisations difficult, and perhaps unwise. In our study, we selected a freshwater species with specialised hearing and a high sensitivity to sound^61,62^. Goldfish may be more reactive at the lower end of the hearing range because they are more sensitive to particle motion at 125–250 Hz^63^. At frequencies below 400 Hz^64^, there may be functional overlap of the neuromast and the otolith^65^. As both the otolith and the neuromast detect particle displacement, the relative contribution of the two to hearing is challenging since the inputs for auditory and lateral line nerves lie in close contact^66^. This means the electrophysiological techniques to measure hearing are likely to detect both inputs^66,67^. However, we would expect this same mechanism to be contributing to the electrophysical audiogram as well as the behavioural methods applied in this study. The importance of particle motion associated with low frequency sound fields has previously been considered in the development of behavioural deterrents, particularly in respect to the protection of European eel (*Anguilla anguilla*)^34,68^.

As the current assumption about the relationship between detection of a sound and response to it^46^ was contradicted by the results reported here, the implications for future fish deterrent design should be considered. It is no longer valid to accept the premise that the frequency of highest probability of reaction corresponds with the greatest sensitive of hearing or that the difference (*ΔThreshold*) between the two remains constant independent of frequency. The observation that *ΔThreshold* varies with frequency supports the consensus within the scientific community^44,45^ that suggest that the continued use of the concept of dB above the hearing threshold (dB_ht_)^43^ used to inform infrastructure projects^69,70^ is inappropriate. The use of an arbitrarily defined fixed level above the basic hearing threshold has been used as a convenient method to set criteria for acceptable sound levels for other animals, such as marine mammals^71^ when knowledge is limited. Our findings support the arguments of others^72–74^ that the logic that underpins the use of dB_ht_ may be flawed, at least in the context described here, and may provide an explanation, at least in part, for why the efficiency of acoustic deterrents can be highly variable^39^.

Moving forward, this study highlights factors that should be further considered in advancing the design of acoustic fish deterrents. First, the importance of behavioural studies in understanding the response of fishes to sound^75^ is reiterated, rather than developing design criteria based on data obtained from physiological methods alone. Quantification of thresholds of reaction is more appropriate from a fisheries management perspective, as avoidance is the often desired response in fish guidance system. Therefore, approaches such as those based on Acoustically Evoked Behavioural Response (AEBR)^60,61^ as used in this study, rather than AEP derived audiograms, are most appropriate because they determine the lowest SPLs over a range of frequencies at which a reaction is elicited. Second, once appropriate frequencies are identified there is a need to select SPLs that evoke the response desired accounting for ambient environmental conditions at the site of interest, i.e. considering appropriate signal-to-noise-ratios. Furthermore, other acoustic parameters should be characterised, including spatial distribution and temporal patterns. Third, this study adopted a novel experimental approach that resolves several of the challenges associated with small tank experiments^76^. By submerging the test cylinder in which the fish were constrained in a large tank, a more homogeneous sound field was created with less boundary reflections that result in unnatural heterogeneity in particle motion and sound pressure. A reductionist experimental approach such as that described here is valuable when there is a need to quantify fine-scale behaviours of the fish in response to rigorously mapped acoustic stimuli while controlling for confounding variables before moving on to field tests of prototype devices. Finally, we recommend that future research is directed at quantifying variability as a result of abiotic (e.g. the hydrodynamic environment^77^) and biotic factors, particularly between species and developmental stage or size^60^ with the view to identifying appropriate representatives guilds of commercial and conservation concern. Likewise, greater understanding is needed of how response to acoustic signals may differ between species that are typically solitary and those that occupy positions within groups (aggregations, shoals and schools^13,30^).

## Methods

### Fish maintenance

Goldfish (n = 80; mean standard length [SD]: 64.0 [5.3] mm; mass: 10.2 [2.4] g) were transported from Hampshire Carp Hatcheries (UK) in oxygenated water to the International Centre for Ecohydraulics Research (ICER) facility, University of Southampton, in November 2020. They were maintained in a holding tank (1.50 m long, 1.00 m wide, and 0.80 m deep, filled to 0.68 m water depth) containing ≈ 1.2 m^3^ of aerated, filtered and dechlorinated water under an artificial photoperiod matching the light levels at the time of year (10:14 h light:dark) and fed once daily (Tetra goldfish flakes; protein: 42%). Ammonia (0.10 [0.22] ppm), nitrites (0 [0] ppm), nitrates (40.0 [0.0] ppm), pH (pH 8.20 [0.00]) (API Freshwater Master Test Kit) and temperature (12.3 [1.2] °C) were monitored daily. Fish were acclimated in the holding tank for at least four days before 12 individuals were selected and moved to the experimental facility (< 700 m) the night prior to the start of trials where they were maintained in a pre-test tank (0.84 m long, 0.5 m wide, and 0.65 m deep, filled to 0.54 m water depth) containing ≈ 0.29 m^3^ of aerated and dechlorinated water for a further 13 h to acclimate to the temperature of the experimental tank (temperature: 13.15 [0.49] °C). On completion of each trial, the subject fish (a single individual per trial) were placed in a post-test tank and returned to a separate holding tank at the ICER Facility at the end of the day.

### Ethical note

Experiments were carried out in compliance with guidelines established by the current UK animal protection law established by the Home Office (Animal Welfare Act 2006). All applicable international, national, and/or institutional guidelines for the care and use of animals were followed. The study was reviewed by the Animal Welfare and Ethics Review Body and approval granted by the University of Southampton Ethics and Research Governance committee (ID: 54900.A1). We reduced the number of goldfish used in our study by using the 25% threshold and we minimised distress by using a relatively low stimulus SPL^78^. Individuals were handled with care, and handling time was kept to a minimum. There was no evidence of stress or fatigue from exposure to the acoustic stimulus in any of the treatments during the trials. The authors complied with the ARRIVE guidelines.

### Experimental setup

**Figure 4:**
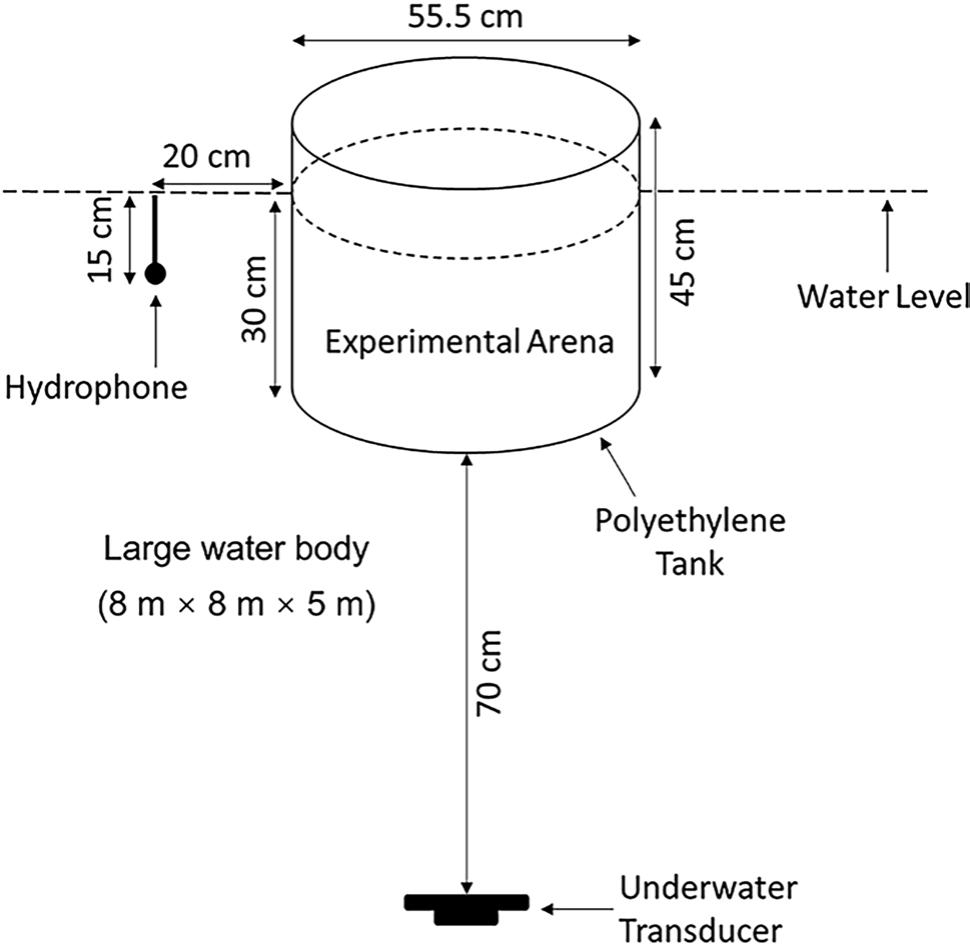
The set-up of an experimental study conducted to investigate the startle reaction of a goldfish in response to 120 ms tones at six frequencies and four sound pressure levels. The fish were constrained within test cylinder positioned within a large tank (8 m width × 8 m length × 5 m depth). The transducer was suspended 70 cm below the tank, and a hydrophone placed 15 cm below the water level (dotted line) at a distance of 20 cm from the cylinder wall.

Trials were conducted in a white medium density polyethylene cylindrical tank (modified 100 L Round Water Tank; 0.55 diameter, 0.41 m deep, 4 mm thick) suspended from a bespoke metal frame into a large water filled tank (8 m long, 8 m wide, 5 m deep) at the A. B. Wood Laboratory, University of Southampton (Fig. 4). The cylinder was submerged in water to resolve the challenges associated with traditional fish hearing experiments that typically use small tanks surrounded by air resulting in sound fields with high boundary reflections. This reduces reverberation caused by the impedance difference between the water in the tank and the surrounding air, resulting in a more realistic and homogeneous sound field. A black polyethylene mesh (6 mm mesh width) covered the tank to prevent escape of leaping fish. The test cylinder was filled to a depth of 30 cm with dechlorinated conditioned water that was replaced (≈ 20 L water change) after each trial to maintain water quality. An underwater transducer (Electro-Voice UW-30; maximal output 153 dB re 1 μPa at 1 m for 150 Hz, Lubell Labs, Columbus, USA) was suspended 0.7 m below the cylinder and a hydrophone (8105, manufacturer-calibrated sensitivity − 205 dB re: 1 V μPa; Brüel & Kjær, Denmark) placed 20 cm from the tank to continuously monitor the sound during each trial. Trials were recorded via a webcam (C920; HD 1080p; 30 frames s^−1^; Logitech Pro, Switzerland) installed directly above the water surface, ensuring that the entire cylinder was maintained within the field of view. The room was lit by fluorescent lighting that provided sufficient illumination for video recording.

### Experimental design

The study consisted of 20 replicates of 24 treatments based on a combination of one of six frequencies (250; 400; 600; 800; 1000; 2000 Hz) and four SPLs (115; 125; 135; 145 dB re 1 μPa) (6 × 4 = 24). Prior to the start of each trial (10–19 November 2020), a single fish (N = 80) was acclimated in the experimental cylinder for 30 min. Fish experienced a total of six exposures (one at each test frequency selected at random) at one of the four randomly assigned test SPLs (see Table 2 for an example). Each exposure consisted of a sinusoidal 120 ms tone ramped with a 20 ms Hanning taper and was followed by 6 min of silence before the next exposure (e.g. Table 2). Although latencies of Mauthner cell activation in goldfish is 5–10 ms, the tone was played at 120 ms to be consistent with the ring-up time of the acoustic system^60,79^. Fish behaviour was continuously video recorded during the trial, and each fish was used in one trial only.

**Table 2:** An example of the selection of the 120 ms pure tone acoustic treatments experienced by an individual goldfish.

Trial	Frequency (Hz)	SPL (dB re 1 μPa)	Order of exposure
1	600	115	1
1	800	145	2
1	400	115	3
1	1000	135	4
1	250	125	5
1	2000	145	6

Trial (n = 80) represents the entire period in which an individual fish experienced the acclimation followed by six treatments. Treatment is each of the 24 combinations of sound pressure level and frequency (n: treatment 1–24). Exposure represents the nth treatment (1–6) experienced by an individual fish in one trial.

### Acoustic stimuli and sound mapping

Sound samples were produced in MATLAB (Release 2019b, The Mathworks, Inc., Natick, USA) using a laptop connected to a DAQ (NI USB-6212; National Instruments, USA), transmitting the signal through an amplifier (Prosound Power AMP 200; frequency response: 20 Hz–20 kHz), and was emitted via the UW30 underwater transducer. Acoustic stimuli were standardised such that the desired SPL was reached in the centre of the experimental arena. Use of artificial stimuli allowed for control of the specific acoustic components tested.

Prior to exposing fish to stimuli, the acoustic environment of the experimental arena was quantified. A total of 246 measurements were made using a calibrated hydrophone (Brüel & Kjær 8105) to produce a 3D representation of the SPL in the experimental arena (Supplementary Fig. S1). The measurements consisted of 82 points, 5 cm apart at depths of 5, 15, and 25 cm measured from the water surface (Table 3). The data capture and stimulus generation were synchronised to facilitate computation of the PA. Both SPL and PA were quantified to create maps of the sound field. The PA, *a*, was calculated as:

1 $$a= -\frac{1}{\rho }\nabla P$$

where *ρ* is the ambient density and *P* is the pressure^80^.

**Table 3:** The mean ± standard deviation of the 145 dB re 1 μPa, 120 ms pure tone (columns 2–5) SPL and particle acceleration (column 6) across the cylindrical tank at frequencies of 250 Hz; 400 Hz; 600 Hz; 800 Hz; 1000 Hz; 2000 Hz.

Frequency (Hz)	5 cm (dB re 1 μPa)	15 cm (dB re 1 μPa)	25 cm (dB re 1 μPa)	Centre SPL (dB re 1 μPa)	15 cm (dB re 1 mm s^−2^)
250	136.3 ± 0.7	143.5 ± 0.6	146.9 ± 0.5	143.3 ± 0.4	18.5 ± 4.8
400	137.9 ± 0.4	145.0 ± 0.5	148.3 ± 0.4	144.9 ± 0.3	19.0 ± 5.1
600	137.5 ± 0.4	144.4 ± 0.4	147.6 ± 0.3	144.5 ± 0.3	20.2 ± 5.0
800	137.2 ± 0.5	144.1 ± 0.5	147.8 ± 0.3	144.2 ± 0.3	22.8 ± 4.1
1000	136.1 ± 0.6	143.2 ± 0.8	145.8 ± 0.3	143.4 ± 0.4	24.5 ± 3.7
2000	139.9 ± 0.8	144.8 ± 0.6	141.0 ± 2.5	145.1 ± 0.1	48.6 ± 4.1

Point measurements were taken at 3 depths (5 cm; 15 cm; 25 cm measured from the water surface). Centre SPL refers to the average of the four SPLs in the middle in the 15 cm layer of the tank.

The pressure gradient was computed using the measurements of the pressure signal. The root mean square (RMS) of the pressure difference was calculated independently in three directions (x, y and z). The pressure gradient was obtained by dividing by the distance between measurements. The RMS PA Eq. (1), in each direction, was calculated by dividing the pressure gradient by the water density. The total RMS PA was determined by combining the values in all three directions, with the results expressed in decibels (dB re 1 mm s^−2^). Following this the PA was represented in maps (Supplementary Fig. S2).

The measured ambient SPL (TC4032, manufacturer-calibrated sensitivity − 170 dB re: 1 V μPa; Teledyne Reson, USA) was on average less than 96 dB re 1 µPa, which was the electrical noise floor of the measurement system being used. The SPL was relatively uniform across the horizontal plane for each frequency, with greatest variation observed at the highest frequency (shortest wavelength) at 2000 Hz (Table 3). The SPL differed by ≈ 10 dB between the top and bottom of the tank. The PA increased with frequency and varied up to 4.8 dB within the horizontal plane.

### Behavioural and data analysis

Video recordings of fish behaviour obtained for each trial were analysed and a startle response defined as a change in body tortuosity with erratic swimming, i.e., a sudden increase in swimming speed or a change in swimming direction^60^. Startles were recorded as present or absent for each trial, thus residuals were modelled using a binomial distribution. Video footage was reviewed blind of the treatment used and in a random order such that 99.5% of the recorded startles were consistent.

All statistical analyses were performed in R (version 3.6.3). Logistic regression was performed using general (GLM) and generalized linear models (GLMM) with a binomial error structure and a “logit” link function. To determine whether external factors may have confounded the results by influencing the probability of startling, a reductive model was developed. Factors included in the model were: tank days (minimum number of days in the holding tank); time (the beginning of the trial to the nearest hour); experimental tank temperature (°C); and size (mass/standard length^2^). The initial GLMM contained all predictor variables with exposure (order of stimulus exposure) and trial included as random effects, and manual backwards selection using variable significance (significance at p < 0.05) was undertaken as model simplification. No random effects were detected (Supplementary Table S2) so fixed variable GLMs were used for further analysis. Exposure and trial were included in a GLM as fixed effects alongside the other external factors in logistic regression, however, none predicted that a startle would occur, and the null model had the optimum AIC (Supplementary Table S3).

Logistic regression was used to determine the influence of SPL and frequency on whether a startle response would occur. Logistic regression curves were, therefore, plotted with SPL against probability of startle for each frequency and the significance of the relationship recorded. The logistic regression was used to determine the 25% deterrence threshold. The 25% threshold was selected based on the principle of ethics in animal research that requires a reduction in the number of individuals used where-ever possible. Hence, the lowest maximum (25%) of the population startled at 600 Hz. The SPL and PA at which 25% of the population startled at each frequency was predicted using the GLM. The output stated the probability that a startle occurred for a stated SPL (or PA). Both the SPL and PA were chosen and refined until the output was within 0.001 or 0.25.

The proportions obtained by prediction using the GLM, quantified as startles per total number of individuals tested at each treatment, were represented on a plot of SPL against frequency and a plot of PA against frequency. Both 1000 Hz and 2000 Hz were omitted since extrapolation was unfeasible due to an insignificant logistic relationship, and predictive models estimated the threshold to be at a level damaging to the ear. The 25% deterrence threshold was plotted on one figure alongside the hearing threshold for goldfish, obtained by averaging AEP sound pressure hearing threshold data from 23 studies (Supplementary Table S1). The mean values of hearing thresholds obtained via AEP were plotted with a 95% confidence interval to allow for comparison between the startle and hearing thresholds. The same analysis was completed for PA and compared to a single threshold calculated using an accelerometer – the most accurate method of measuring PA hearing ability^63^.

The difference between the deterrence and the hearing thresholds were calculated and followed a Gaussian distribution, determined by visual inspection of the qq curve. The *ΔThreshold* was defined as the difference between thresholds for hearing and 25% startle response. The sound pressure *ΔThreshold* was calculated by subtracting published AEP hearing threshold values from the 25% deterrence threshold at each frequency. The same method was used for the PA *ΔThreshold* such that the published PA hearing threshold values were subtracted from the 25% deterrence threshold at each frequency. To assess the influence of frequency on threshold range, a GLM with Gaussian error structure was applied for both SPL and PA *ΔThreshold*. Planned orthogonal comparisons were used to determine whether the SPL *ΔThreshold* at 250, 400, 600 and 800 Hz differed from each other. This was not undertaken for PA, as there was only a single data point for each frequency since the PA threshold was based on a single study.

### Supplementary Information

Supplementary Information.

## Data Availability

Supplementary data supporting this study are openly available from the University of Southampton repository (University of Southampton, 2022) at https://doi.org/10.5258/SOTON/D2486.
