# Author Correction: Applying appropriate frequency criteria to advance acoustic behavioural guidance systems for fish

**DOI:** 10.1038/s41598-024-56278-w

**Published:** 2024-03-06

**Authors:** A. Holgate, P. R. White, T. G. Leighton, P. S. Kemp

**Affiliations:** 1 International Centre for Ecohydraulics Research, Faculty of Engineering and Physical Sciences, University of Southampton, Southampton, UK; 2 Institute of Sound and Vibration Research, Faculty of Engineering and Physical Sciences, University of Southampton, Southampton, UK

Correction to: *Scientific reports* 10.1038/s41598-023-33423-5, published online 18 May 2023

The original version of this Article contained an error in Figure 4, where the dimensions of the large water-filled tank were incorrect.

The original Figure 4 and accompanying legend appear below.

**Figure 4:**
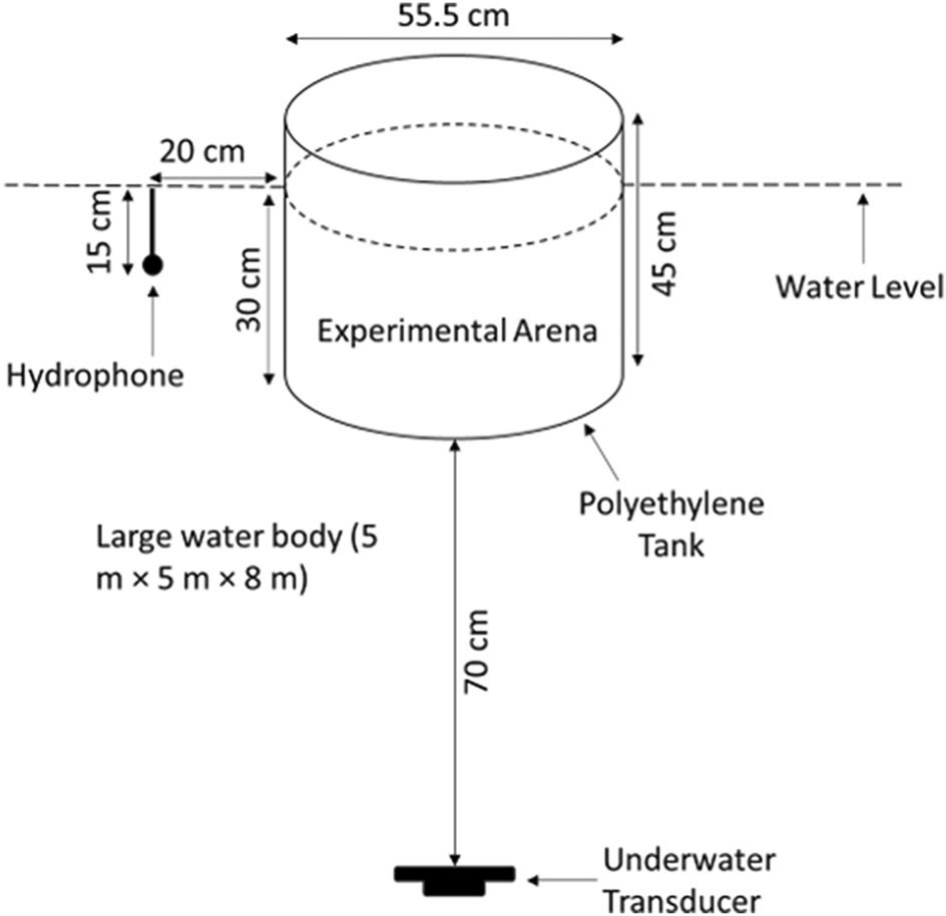
The set-up of an experimental study conducted to investigate the startle reaction of a goldfish in response to 120 ms tones at six frequencies and four sound pressure levels. The fish were constrained within test cylinder positioned within a large tank (8 m width × 8 m length × 5 m depth). The transducer was suspended 70 cm below the tank, and a hydrophone placed 15 cm below the water level (dotted line) at a distance of 20 cm from the cylinder wall.

The original Article has been corrected.

