# Supplementary material for: Applying appropriate frequency criteria to advance acoustic behavioural guidance systems for fish
Source: Sci Rep. 2023 May 18;13:8075. doi: 10.1038/s41598-023-33423-5 (PMC10195784; doi:10.1038/s41598-023-33423-5)
Supplement: Supplementary file 1 — Supplementary Information. [file 41598_2023_33423_MOESM1_ESM.docx]

Supplementary Table S1.

| Author | Year | 200 Hz | 250 Hz | 300 Hz | 400 Hz | 500 Hz | 600 Hz | 700 Hz | 800 Hz | 900 Hz | 1000 Hz | 1500 Hz | 2000 Hz |
| --- | --- | --- | --- | --- | --- | --- | --- | --- | --- | --- | --- | --- | --- |
| Amoser | 2003 | 74.5 | **71.4** | 68.3 | **65.55** | 62.8 | **63.413** |  | 64.7 |  | 66.1 |  | 81.3 |
| Cordova | 2007 | 90.3 | **88.375** | **86.45** | 82.6 |  | 80.9 |  | 81.3 |  | 76.5 | 90.1 | **93.7** |
| Gutscher | 2011 | **81.25** | **77.62** | 75.2 | **71.6** | 68 | **66.313** |  | 63 |  | 69.2 |  | 97.7 |
| Kenyon | 1998 | 73.3 | **71.05** | 68.8 | 63.9 | 64 | 64.1 |  | 64 |  | 64.6 | 71.5 | 80 |
| Kojima | 2005 | 72.7 | **70.55** | 68.4 | 63.6 | 63.5 | 63.5 |  | 63.6 |  | 64.2 | 70.7 | 79.3 |
| Ladich | 1999 | 71.9 | **69.925** |  | 64 |  | **64.147** |  | **64.337** |  | 64.5 |  | 78.3 |
| Lu | 2002 | 83.8 | **82.4** |  | **79.147** | 76.8 | **77.4** |  | **78.6** |  | 79.8 |  | 106 |
| Ramcharitar | 2006 | 84 | **82.5** | 81 | 73 | 71.5 | 67.5 | 70.4 | 67.6 | 68.5 | 74.4 |  | 77.9 |
| Ramcharitar | 2010 | 63.4 | **62.4** | 61.4 | 71.1 | 77.2 | 75.5 | 86.9 | 81.7 | 92.4 | 85.1 |  | 85.9 |
| Smith | 2004a | 89.1 | **83.85** |  | 68.1 |  | 74.7 |  | 74.4 |  | 79.8 |  | 97.2 |
| Smith | 2004b | 79.4 | **78.3** |  | 75 |  | 75.3 |  | 78.4 |  | 79.2 |  | 90.3 |
| Smith | 2006 | 101.6 | **97.1** |  | 83.6 |  | 79 |  | 80 |  | 79.3 |  | 104.1 |
| Smith | 2011 | **84.027** | 82.7 |  | 76.8 | 75.9 | 65.4 |  | 65.9 |  | 64 | 72.6 | 93 |
| Suga | 2005 |  | 87.4 |  | **84.1** | 81.9 | **82.88** |  | **84.84** |  | 86.8 |  | 93.4 |
| Wysocki | 2005 | 76.3 | **73.98** |  | **68.553** | 64.7 | 65.14 |  | **66.02** |  | 66.9 |  | 85.9 |
| Yan | 2000 |  |  | 69.1 | **66.8** | 64.5 | **64.4** |  | 64.2 |  | **66.414** | 71.8 | **86** |
|  |  |  |  |  |  |  |  |  |  |  |  |  |  |
|  |  |  |  |  |  |  |  |  |  |  |  |  |  |
| AEP avg |  | 75.29153 | 77.2048 | 71.07 | 70.05938 | 70 | 69.26831 | 79.35 | 71.46813 | 83.06667 | 73.6125 | 82.3125 | 90.81456 |
| AEP sd |  | 8.051397 | 11.12149 | 10.29867 | 9.056757 | 9.904544 | 9.311813 | 6.808451 | 10.3274 | 10.4353 | 9.424826 | 15.48188 | 14.75259 |
| AEP Med |  | 74.5 | 76.14 | 68.6 | 68.9265 | 68.1 | 67.0265 | 80.75 | 66.81 | 88.3 | 75.4 | 81.85 | 89.65 |

Table S1. Hearing threshold (Sound Pressure Level – SPL) data (dB re 1 μPa) for goldfish (*Carassius auratus*) using the auditory evoked potential (AEP) hearing method at 12 frequencies (200; 250; 300; 400; 500; 600; 700; 800; 900; 1,000; 1,500; 2,000 Hz). Author: Lead author; Year: publishing year; AEP avg: The average hearing level at each frequency (dB); AEP sd: The standard deviation at each frequency (dB); AEP med: The median hearing level at each frequency (dB). Values in bold were calculated by averaging and scaling the hearing level at the two closed frequencies.

Table S1 References:

Amoser, S., and Ladich, F. (2003). Diversity in noise-induced temporary hearing loss in otophysine fishes. *The Journal Of The Acoustical Society Of America*, 113(4), pp.2170-2179. doi: 10.1121/1.1557212.

Cordova, M. S. and Braun, C. B. (2007). The use of anesthesia during evoked potential audiometry in goldfish (Carassius auratus). *Brain Research,* 1153, pp.78-83. doi: 10.1016/j.brainres.2007.03.055.

Gutscher, M., Wysocki, L., and Ladich, F. (2011). Effects of aquarium and pond noise on hearing sensitivity in an otophysine fish. *Bioacoustics*, 20(2), pp.117-136. doi: 10.1080/09524622.2011.9753639.

Kenyon, T., Ladich, F. and Yan, H. (1998). A comparative study of hearing ability in fishes: the auditory brainstem response approach. *Journal of Comparative Physiology A: Sensory, Neural, and Behavioral Physiology*, 182(3), pp.307-318. doi: 10.1007/s003590050181.

Kojima, T., Ito, H., Komada, T., Taniuchi, T., and Akamatsu, T. (2005). Measurements of auditory sensitivity in common carp Cyprinus carpio by the auditory brainstem response technique and cardiac conditioning method. *Fisheries Science*, 71(1), pp.95-100. doi: 10.1111/j.1444-2906.2005.00935.x

Ladich, F. (1999). Did Auditory Sensitivity and Vocalization Evolve Independently in Otophysan Fishes?. *Brain, Behavior And Evolution*, 53(5-6), pp.288-304. doi: 10.1159/000006600.

Lu, Z., and Tomchik, S. (2002). Effects of a red-tide toxin on fish hearing. *Journal Of Comparative Physiology A: Sensory, Neural, And Behavioral Physiology*, 188(10), pp.807-813. doi: 10.1007/s00359-002-0369-8.

Ramcharitar, J., and Selckmann, G. (2010). Differential ablation of sensory receptors underlies ototoxin-induced shifts in auditory thresholds of the goldfish (Carassius auratus). *Journal Of Applied Toxicolo*gy, 30(6), pp.536-541. doi: 10.1002/jat.1523.

Ramcharitar, J. U., Higgs, D. M., and Popper, A. N. (2006). Audition in sciaenid fishes with different swim bladder-inner ear configurations. *The Journal Of The Acoustical Society Of America*, 119, pp.439–443. doi: 10.1121/1.2139068.

Smith, M., Kane, A. S., and Popper, A. N. (2004a). Noise-induced stress response and hearing loss in goldfish (Carassius auratus). *Journal Of Experimental Biology*, 207(3), pp.427-435. doi: 10.1242/jeb.00755.

Smith, M., Kane, A. S., and Popper, A. N. (2004b). Acoustical stress and hearing sensitivity in fishes: does the linear threshold shift hypothesis hold water?. *Journal Of Experimental Biology*, 207(20), pp.3591-3602. doi: 10.1242/jeb.01188.

Smith, M., Coffin, A., Miller, D., and Popper, A. (2006). Anatomical and functional recovery of the goldfish (Carassius auratus) ear following noise exposure. *Journal Of Experimental Biology*, 209(21), pp.4193-4202. doi: 10.1242/jeb.02490.

Smith, M., Schuck, J., Gilley, R., and Rogers, B. (2011). Structural and functional effects of acoustic exposure in goldfish: evidence for tonotopy in the teleost saccule. *BMC Neuroscience*, 12(1). doi: 10.1186/1471-2202-12-19.

Suga, T., Akamatsu, T., Kawabe, R., Hiraishi, T., and Yamamoto, K. (2005). Method for underwater measurement of the auditory brainstem response of fish. *Fisheries Science*, 71(5), pp.1115-1119. doi: 10.1111/j.1444-2906.2005.01071.x

Wysocki, L., and Ladich, F. (2005). Hearing in Fishes under Noise Conditions*. Journal Of The Association For Research In Otolaryngology*, 6(1), 28-36. doi: 10.1007/s10162-004-4043-4.

Yan, H., Fine, M., Horn, N., and Colon, W. (2000). Variability in the role of the gasbladder in fish audition. Journal Of Comparative Physiology A: Sensory, Neural, And Behavioral Physiology, 186(5), pp.435-445. doi: 10.1007/s003590050443.

Supplementary Fig. S1

Sound pressure level (SPL) maps of the submerged arena. The speaker was placed 70 cm below the tank and played at 250 Hz; 400 Hz; 600 Hz; 800 Hz; 1,000 Hz; 2,000 Hz. The SPL was mapped, and the SPL was calibrated at 145 dB re 1 µPa in the centre of the arena. Each figure is titled by the frequency played and the depth of recording.


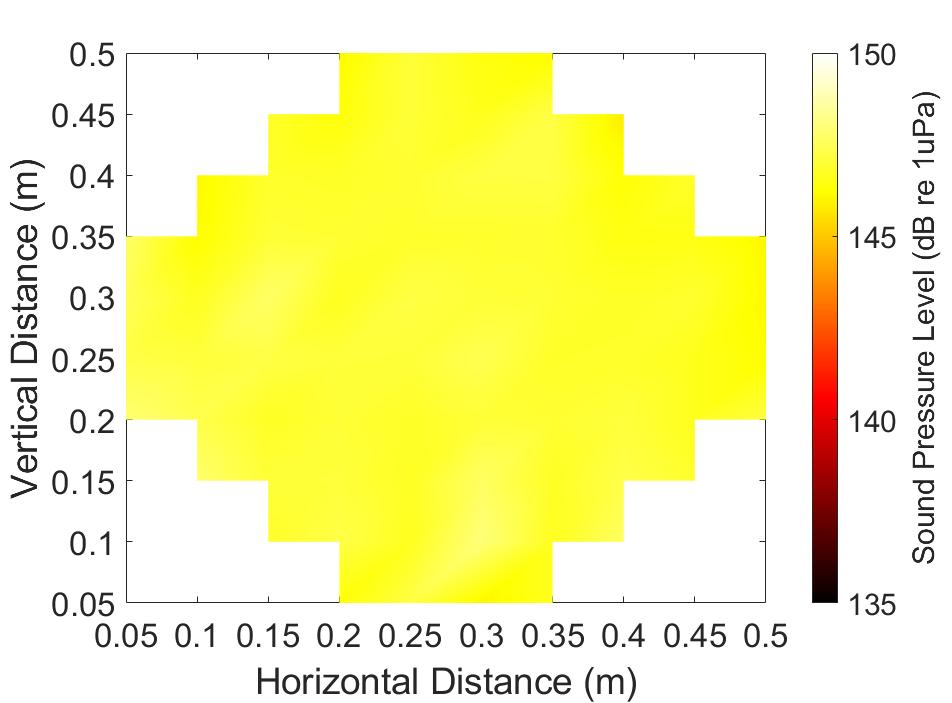

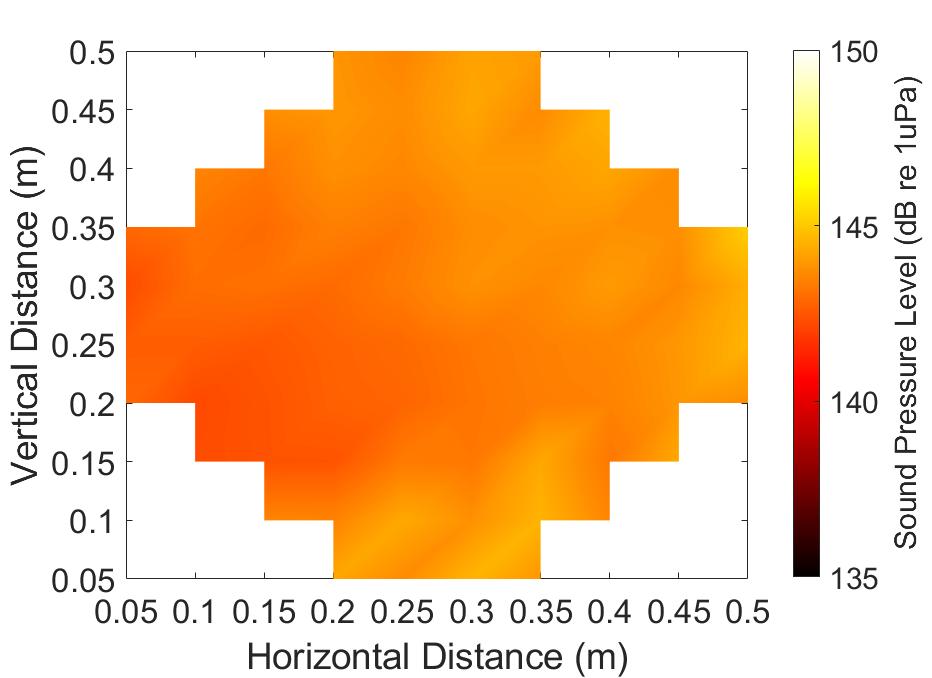

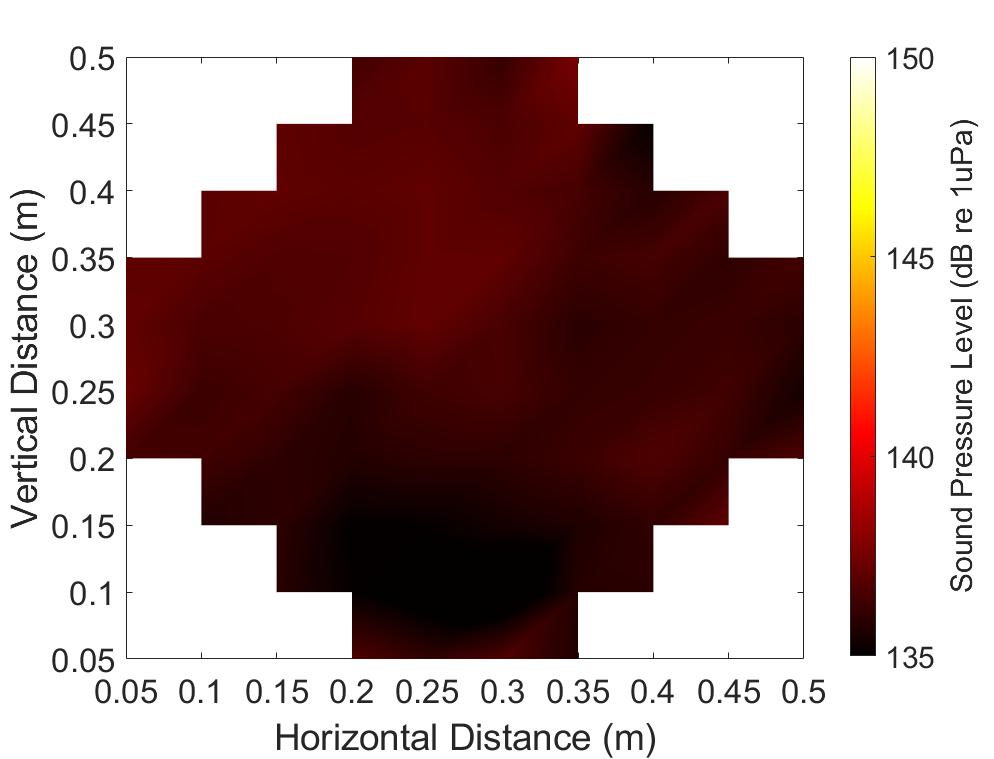


250 Hz: 25 cm

250 Hz: 15 cm

250 Hz: 5 cm


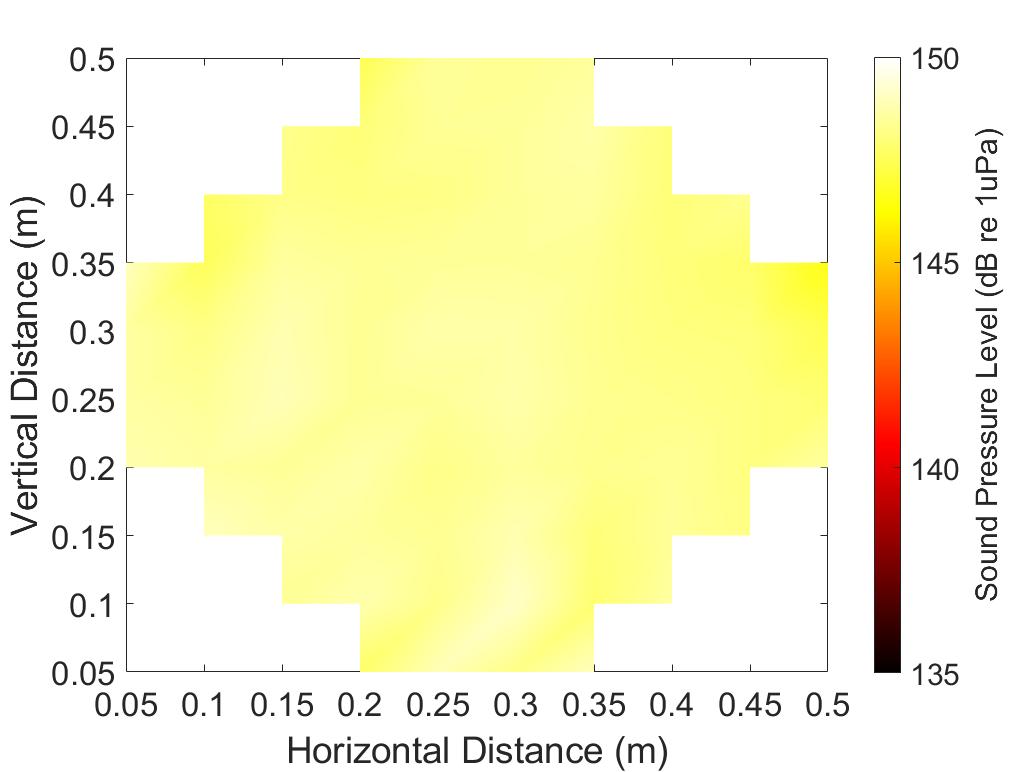

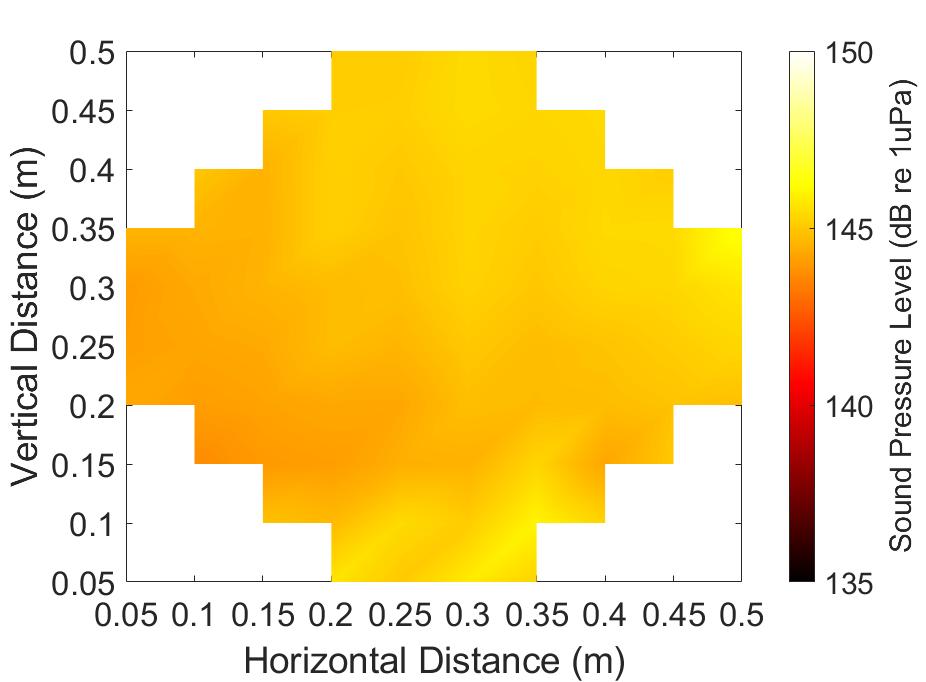

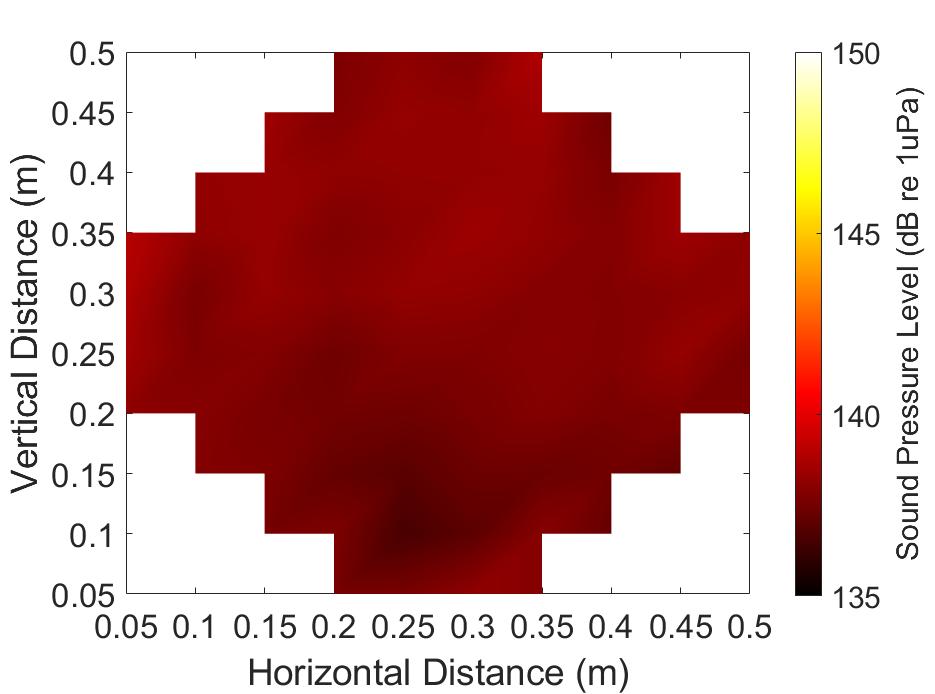


400 Hz: 25 cm

400 Hz: 15 cm

400 Hz: 5 cm


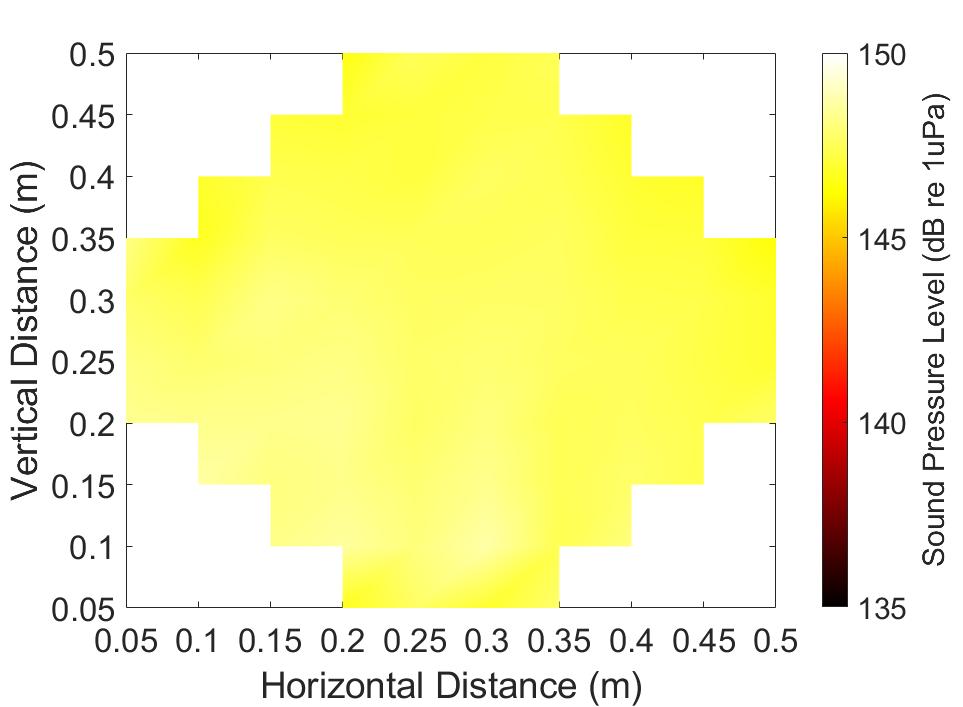

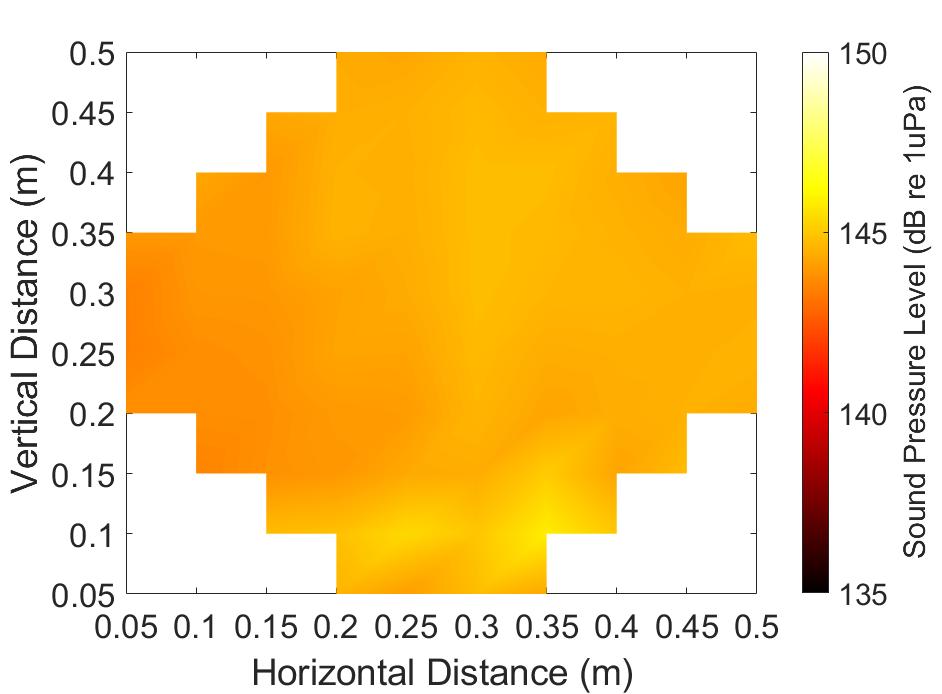

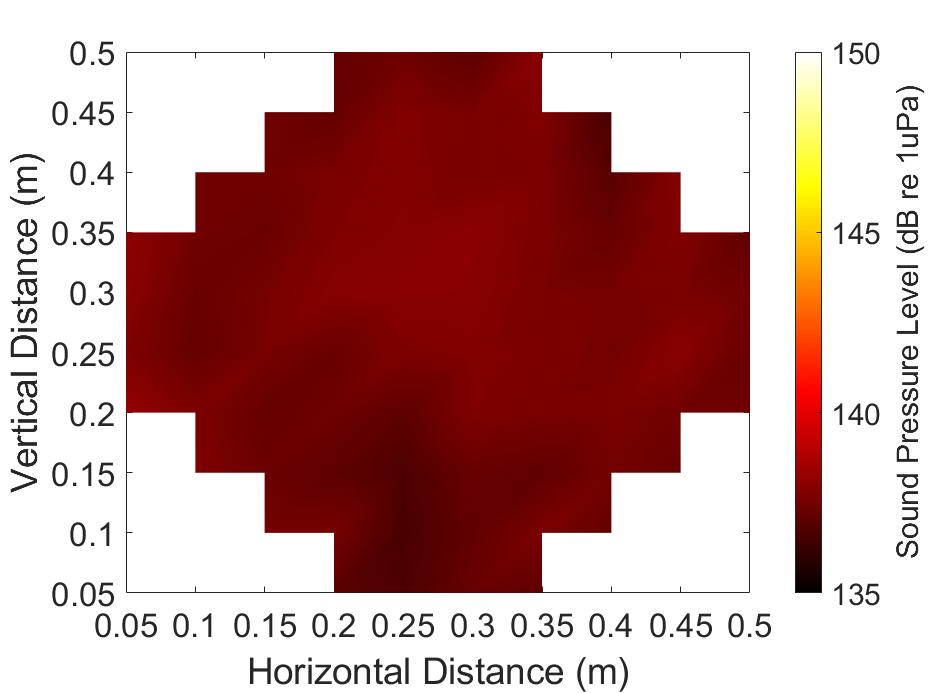


600 Hz: 15 cm

600 Hz: 25 cm

600 Hz: 5 cm


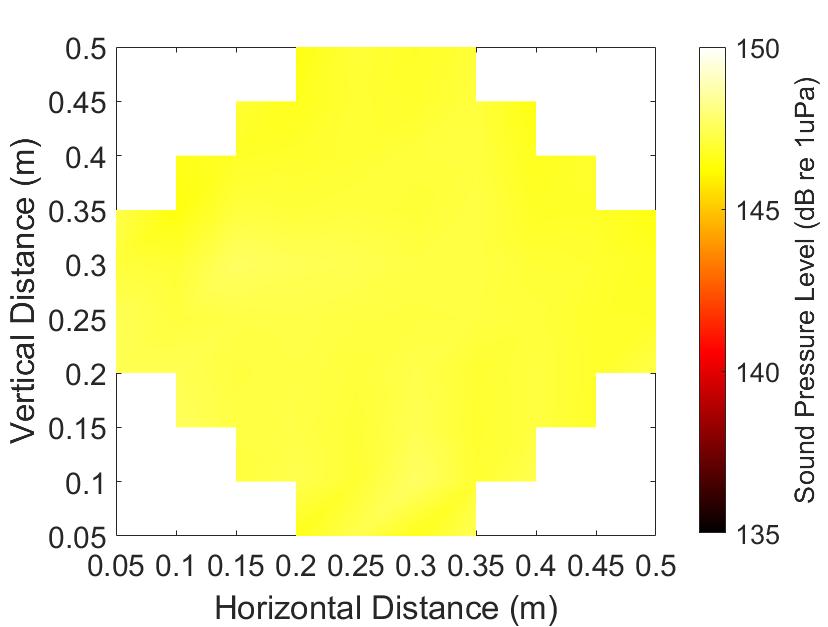

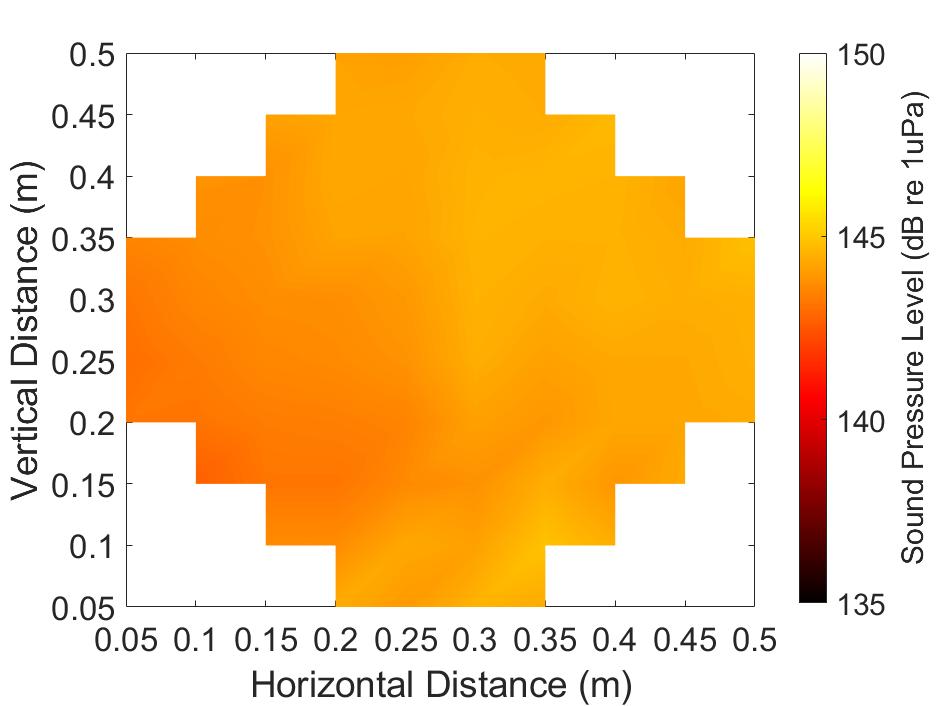

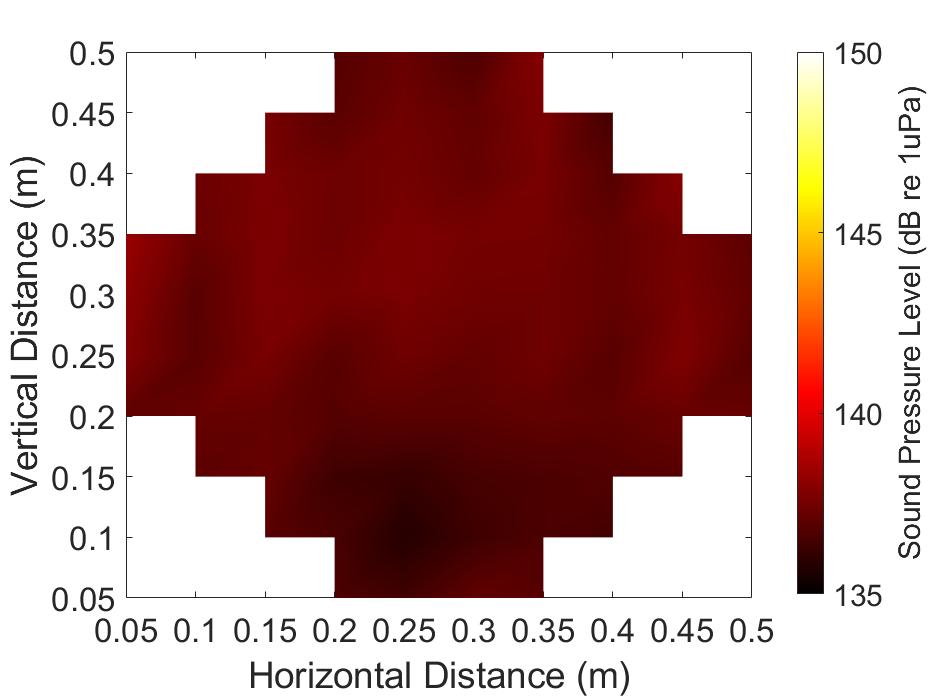


800 Hz: 25 cm 25 cm

800 Hz: 15 cm 25 cm

800 Hz: 5 cm 25 cm


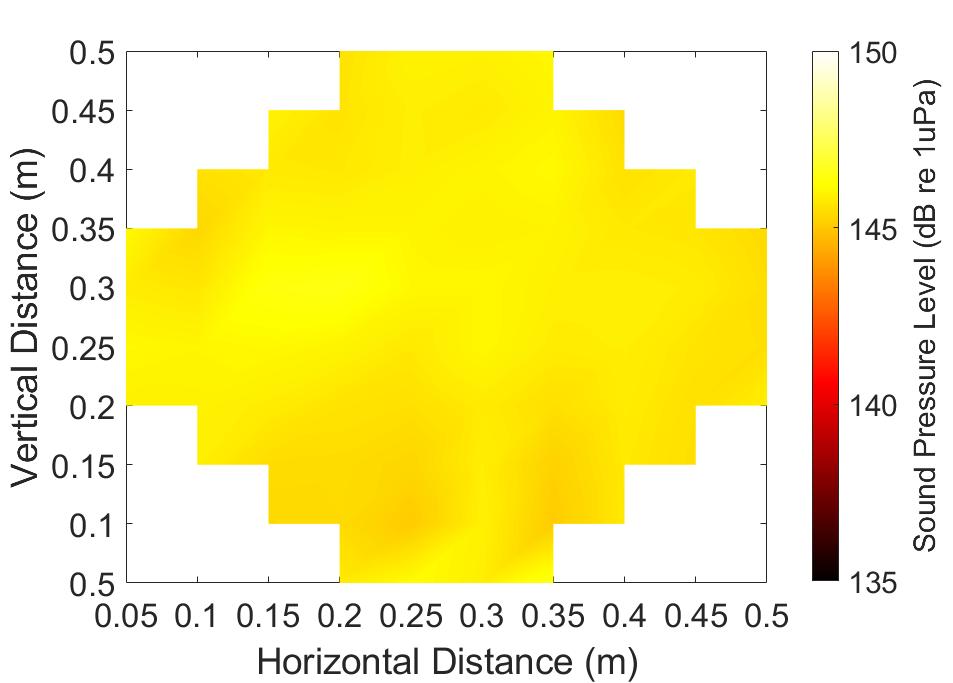

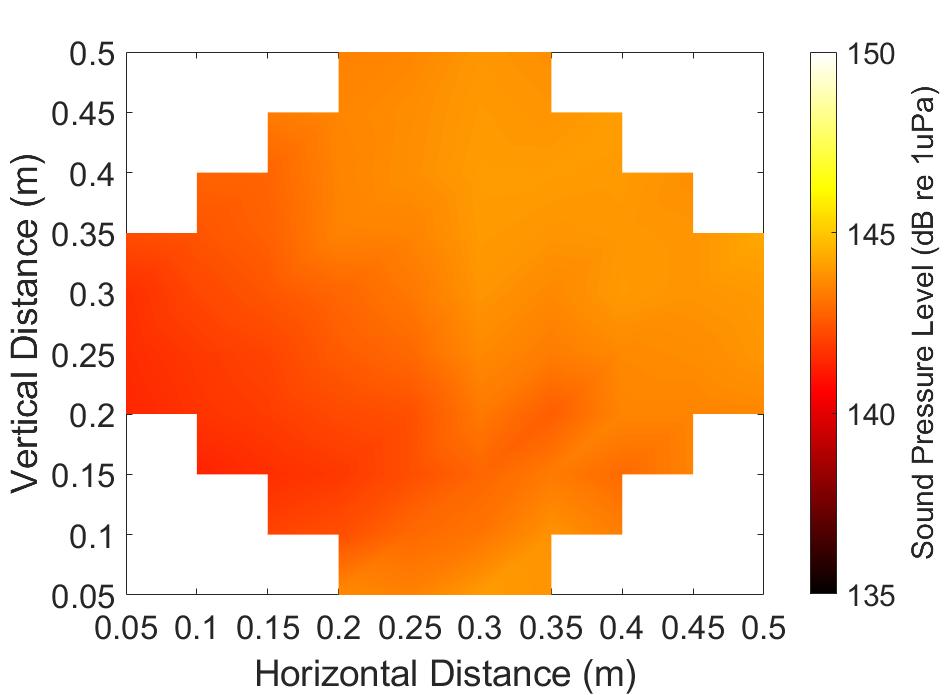

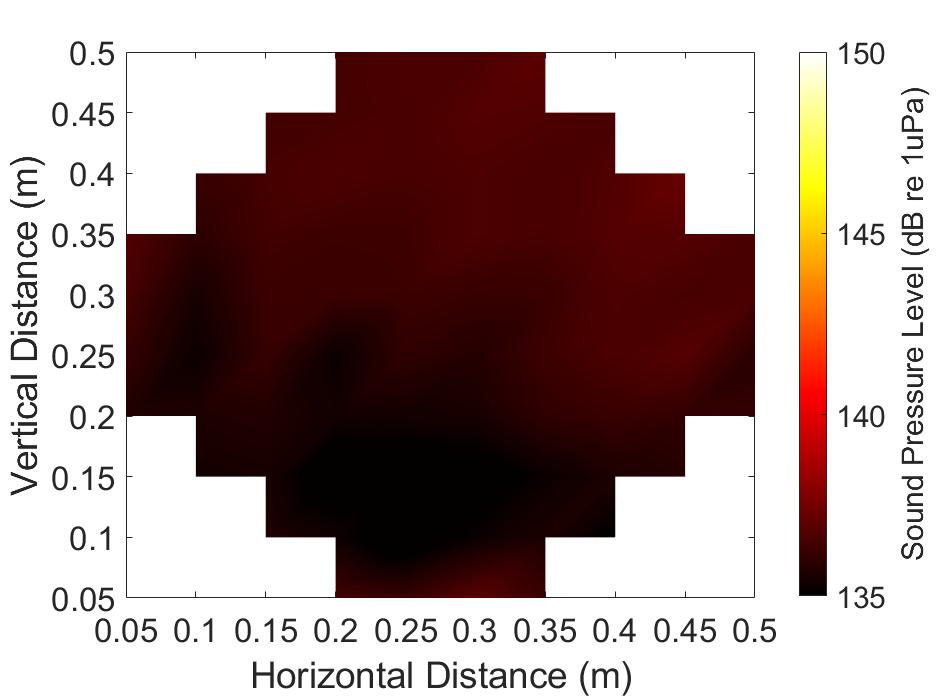


1000 Hz: 25 cm 25 cm

1000 Hz: 15 cm 25 cm

1000 Hz: 5 cm 25 cm


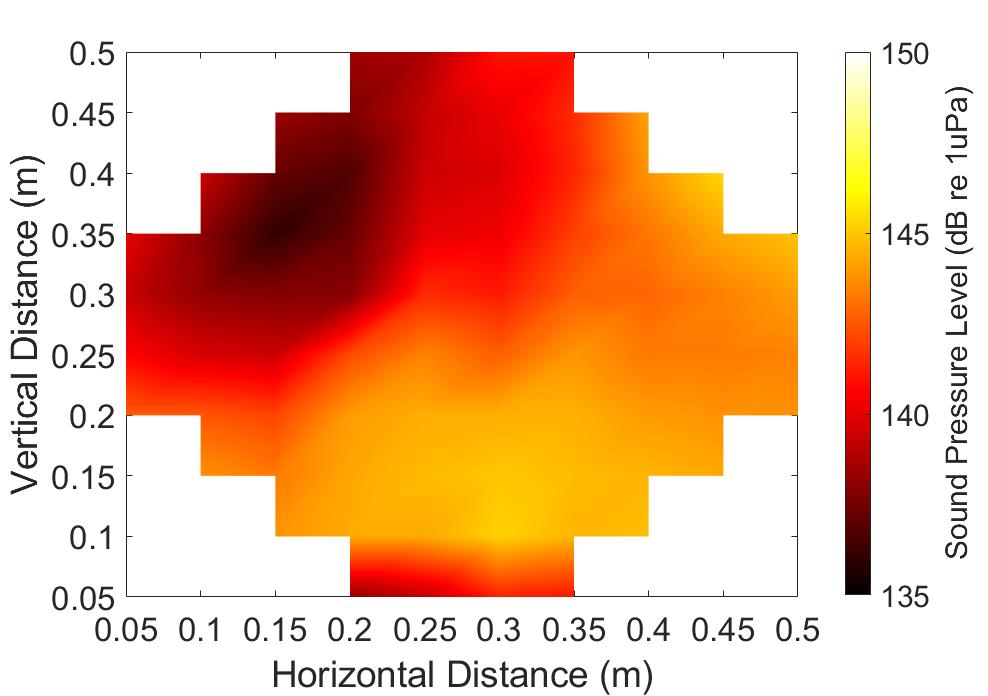

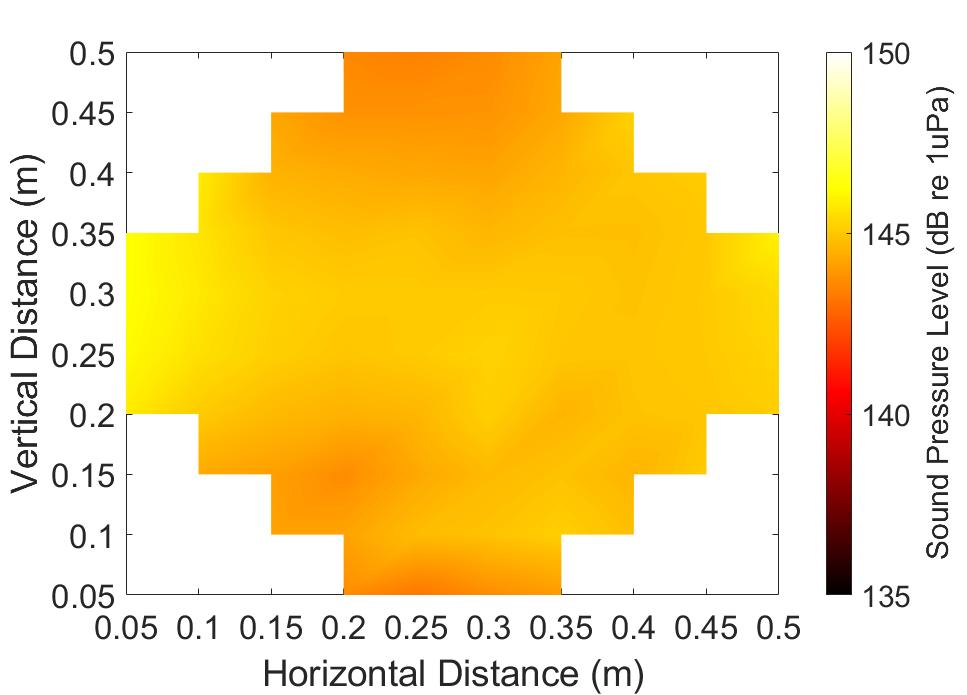

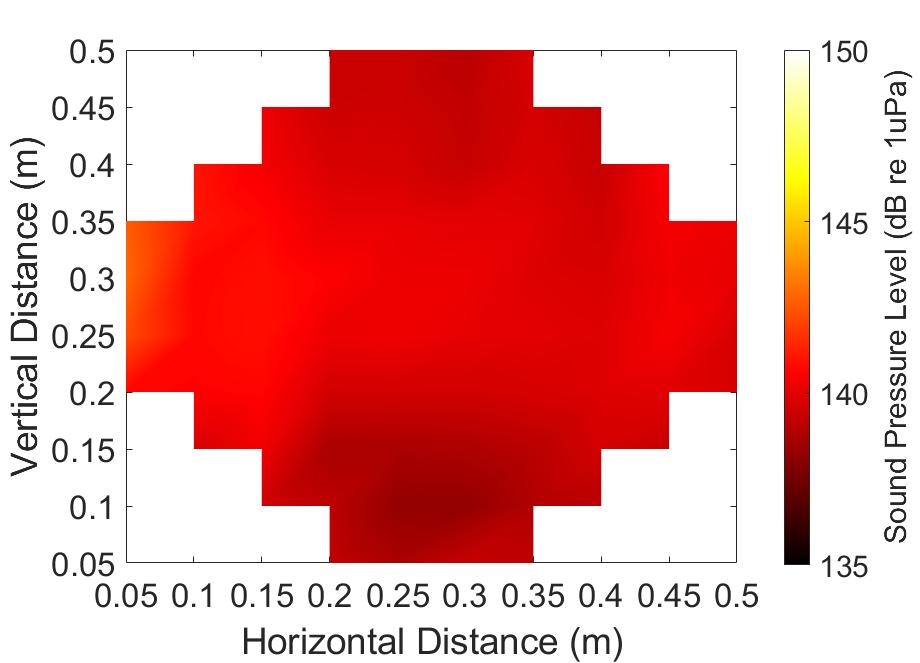


2000 Hz: 25 cm 25 cm

2000 Hz: 15 cm 25 cm

2000 Hz: 5 cm 25 cm

Supplementary Fig. S2 Particle acceleration (PA) maps of the submerged arena at 15 cm depth. The speaker was placed 70 cm below the tank and played at 250 Hz; 400 Hz; 600 Hz; 800 Hz; 1,000 Hz; 2,000 Hz. The SPL was calibrated at 145 dB re 1 µPa in the centre of the arena and PA was calculated (see Holgate et al., for calculation). Each figure is titled by the frequency.


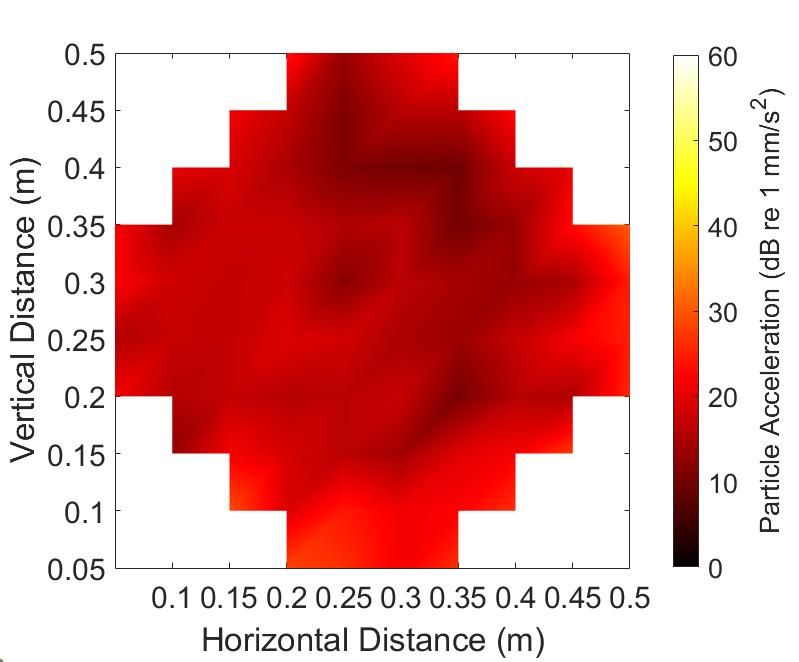

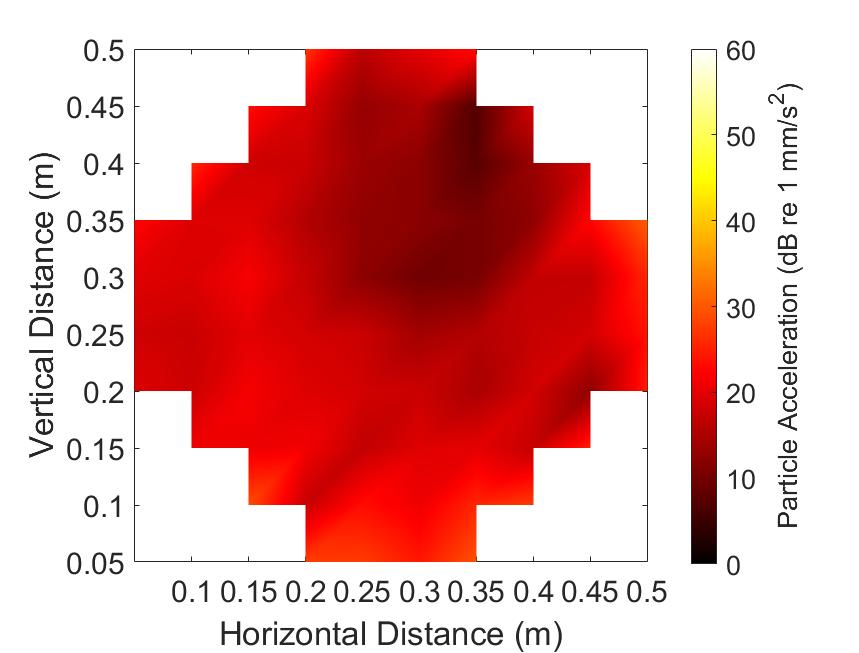

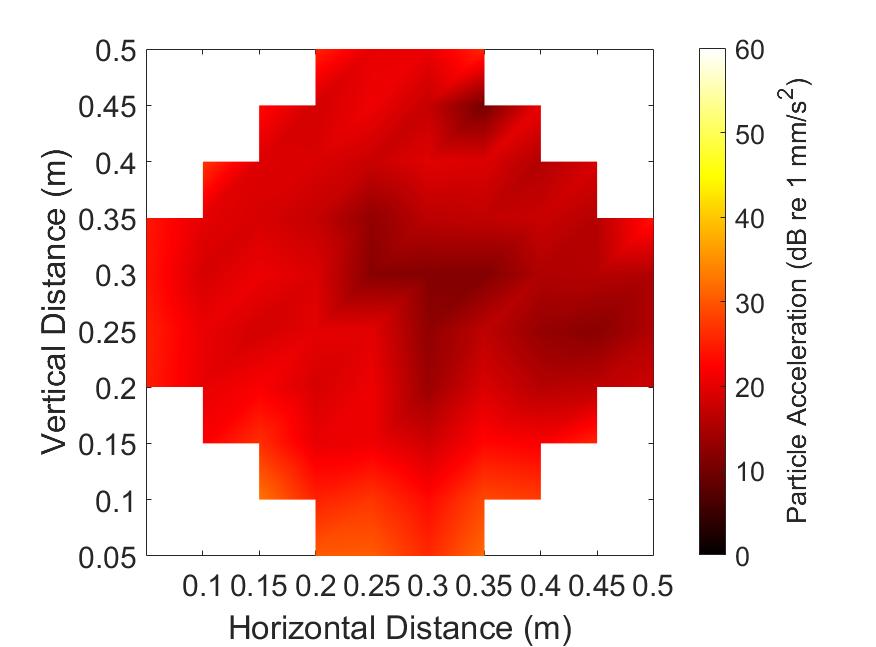


400 Hz

250 Hz

600 Hz


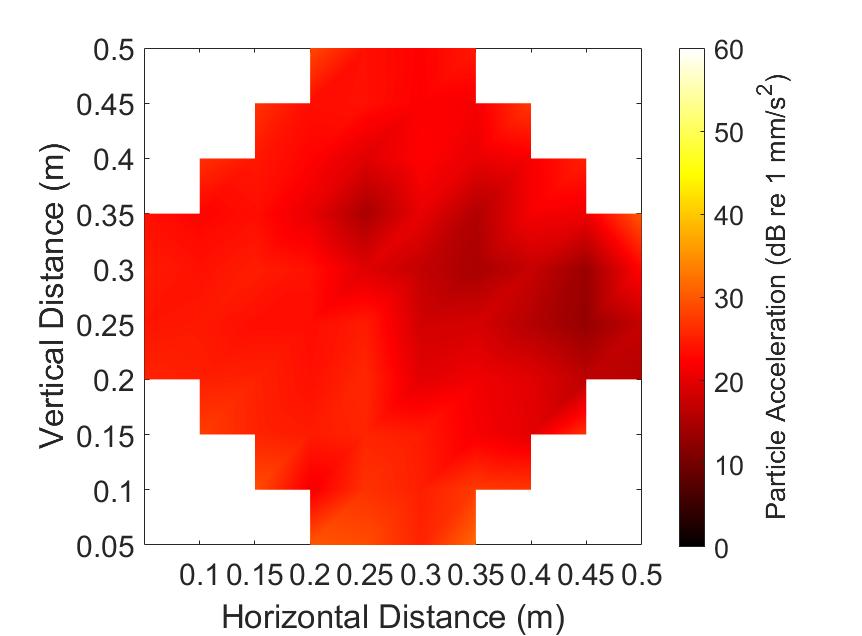

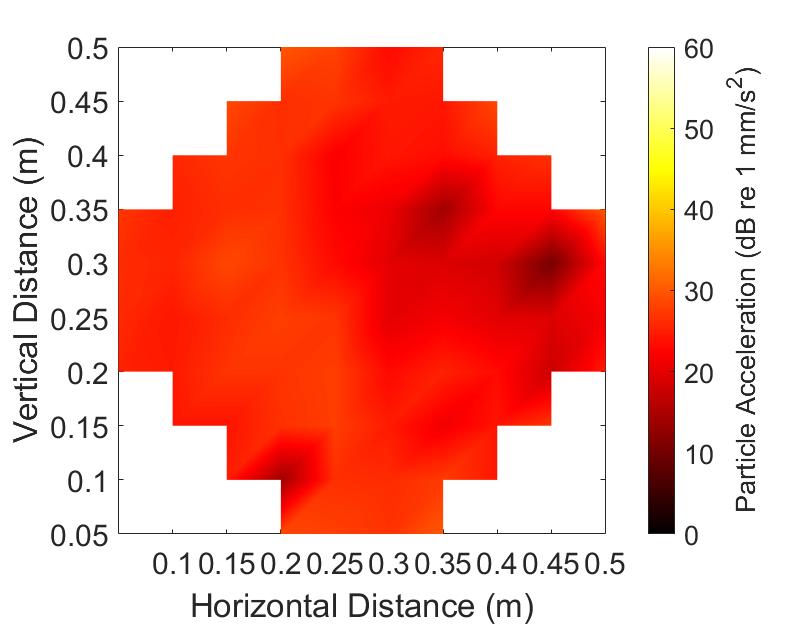

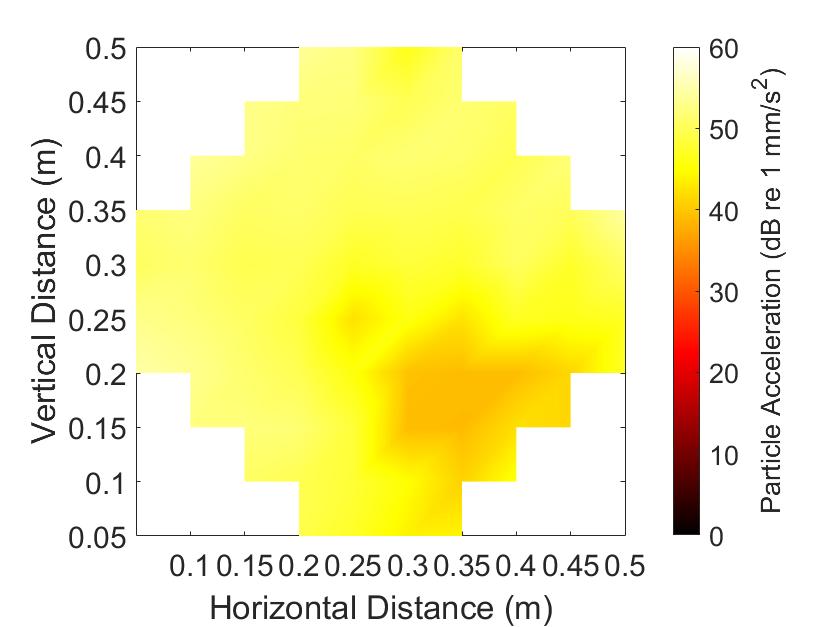


1000 Hz

800 Hz

2000 Hz

**Supplementary Table S2**

Random effects included in generalized linear models developped to determine if external factors affect the presence of a startle response to 120 ms pure tones (treatments of 250, 400, 600, 800, 1000, 2000 Hz and 115, 125, 135, 145 dB re 1 μPa) in goldfish.

| ***Random Effect*** | ***Std. dev*** | ***AIC*** |
| --- | --- | --- |
| Null | - | 506.31 |
| Exposure:Trial | <0.001 | 510.3 |
| Trial | 0 | 508.3 |
| Exposure | <0.001 | 508.3 |

**Supplementary Table S3**

Variables included in generalised linear models developped to determine if external factors affect the presence of a startle response to 120 ms pure tones (treatments of 250, 400, 600, 800, 1000, 2000 Hz and 115, 125, 135, 145 dB re 1 μPa) in goldfish.

|  | ***Variables in Model^b^*** | | | |  |  |
| --- | --- | --- | --- | --- | --- | --- |
| ***Model^a^*** | ***Exposure*** | ***Tank Days*** | ***Time*** | ***Temp*** | ***AIC*** | ***ΔAIC*** |
| Null |  |  |  |  | 506.31 | 0.00 |
| 1 | x | x | x | x | 511.57 | 5.26 |
| 2 | x | x | x |  | 509.61 | 3.30 |
| 3 |  | x | x |  | 508.07 | 1.76 |
| 4 |  |  | x |  | 506.86 | 0.55 |

^a^ The null model is shown without any predictor variables and only models with ΔAIC < 7 are shown [Burnham and Anderson, 2002].

*^b^ Exposure (the nth stimulus played 1-6); tank days (minimum number of days in the husbandry tank); time (the beginning of the trial to the nearest hour); temp (experimental tank temperature, °C).*
